# Activation of CXCR3^+^ Tfh cells and B cells in lymph nodes during acute HIV-1 infection correlates with HIV-specific antibody development

**DOI:** 10.1128/jvi.01532-24

**Published:** 2025-02-11

**Authors:** Julie L. Mitchell, Supranee Buranapraditkun, Pierre Gantner, Hiroshi Takata, Kenneth Dietze, Kombo F. N'guessan, Justin Pollara, Junsuke Nohara, Roshell Muir, Eugene Kroon, Suteeraporn Pinyakorn, Nicha Tulmethakaan, Sopark Manasnayakorn, Suthat Chottanapund, Pattarawat Thantiworasit, Peeriya Prueksakaew, Nisakorn Ratnaratorn, Suwanna Puttamaswin, Bessara Nuntapinit, Lawrence Fox, Elias K. Haddad, Dominic Paquin-Proulx, Praphan Phanuphak, Carlo P. Sacdalan, Nittaya Phanuphak, Jintanat Ananworanich, Denise Hsu, Sandhya Vasan, Guido Ferrari, Nicolas Chomont, Lydie Trautmann, on behalf of RV254 and RV304 Study Groups

**Affiliations:** 1 Vaccine and Gene Therapy Institute, Oregon Health & Science University 56870 , Beaverton, Oregon, USA; 2 Henry M. Jackson Foundation for the Advancement of Military Medicine, Inc. 44069 , Bethesda, Maryland, USA; 3 U.S. Military HIV Research Program, Walter Reed Army Institute of Research 8394 , Silver Spring, Maryland, USA; 4 Department of Medicine, Faculty of Medicine, Chulalongkorn University 65103 , Bangkok, Thailand; 5 Center of Excellence in Vaccine Research and Development, Faculty of Medicine, Chulalongkorn University 65103 , Bangkok, Thailand; 6 Thai Pediatric Gastroenterology, Hepatology and Immunology (TPGHAI) Research Unit, Faculty of Medicine, Chulalongkorn University 65103 , Bangkok, Thailand; 7 Centre de Recherche du CHUM (CRCHUM) and Department of Microbiology, Infectiology and Immunology, Université de Montréal 236710 , Montreal, Québec, Canada; 8 Department of Surgery, Duke University Medical Center 609772 , Durham, North Carolina, USA; 9 Division of Infectious Diseases & HIV Medicine, Department of Medicine, Drexel University College of Medicine 12312 , Philadelphia, Pennsylvania, USA; 10 SEARCH Research Foundation, Bangkok, Thailand; 11 Department of Surgery, Faculty of Medicine, Chulalongkorn University 625401 , Bangkok, Thailand; 12 Armed Forces Research Institute of Medical Sciences in Bangkok 19965 , Bangkok, Thailand; 13 Division of AIDS, National Institute of Allergy and Infectious Diseases, National Institutes of Health 35037 , Bethesda, Maryland, USA; 14 Research Affairs, Faculty of Medicine, Chulalongkorn University 65103 , Bangkok, Thailand; 15 Institute of HIV Research and Innovation (IHRI) 606508 , Bangkok, Thailand; 16 Department of Global Health, Amsterdam University Medical Centers, University of Amsterdam 1234 , Amsterdam, the Netherlands; 17 Amsterdam Institute for Global Health and Development 392520 , Amsterdam, the Netherlands; University Hospital Tübingen, Tübingen, Germany

**Keywords:** human immunodeficiency virus, Tfh cells, B-cell responses, humoral immunity, lymph node

## Abstract

Lymph node T follicular helper (Tfh) cells and germinal center (GC) B cells are critical to generate potent antibodies but are rarely possible to study in humans. To understand how Tfh/GC B-cell interactions during acute HIV-1 infection (AHI) impact the generation of HIV-specific antibodies, we performed a unique cross-sectional analysis of inguinal lymph node biopsies taken prior to antiretroviral therapy (ART) initiation in AHI. Although total Tfh and GC B cell frequencies did not change during AHI, increased frequencies of proliferating Th1-like CXCR3^+^ Tfh, CXCR3^+^ non-GC B cells, and total CXCR3^+^ GC B cells correlated with gp120-specific IgG antibody levels in AHI. Frequencies of proliferating CXCR3^+^ Tfh in AHI also correlated with gp120-specific IgG antibody levels after 48 weeks of ART, antibody-dependent cellular cytotoxicity, antibody-dependent cellular phagocytosis, and increased antibody binding to infected cells after ART. Importantly, while beneficial for antibody development, CXCR3^+^ Tfh cells were also infected by HIV-1 at higher frequencies than their CXCR3^−^ counterparts and may contribute to the initial dissemination of HIV-1 in follicles. Together, these data suggest that activation of CXCR3^+^ Tfh cells is associated with induction of the germinal center response and subsequent antibody development, making these cells an important target for future therapeutic interventions.

**IMPORTANCE:**

Early initiation of antiretroviral therapy (ART) is important to limit the seeding of the long-lasting HIV-1 reservoir; however, it also precludes the development of HIV-specific antibodies that can help control the virus if ART is stopped. Antibody development occurs within germinal centers in the lymph node and requires activation of both antigen-specific B cells and T follicular helper cells (Tfh), a specialized CD4^+^ cell that provides B cell help. To understand how early ART initiation may prohibit antibody development, we analyzed the frequencies and activation status of Tfh and B cells in lymph node biopsies collected in the different stages of acute HIV-1 infection. Our data suggest that decreased antibody development after early ART initiation may be due to limited germinal center development at the time of treatment and that new interventions that target activation of CXCR3^+^ Tfh may be beneficial to increase long-term HIV-specific antibody levels.

## INTRODUCTION

The antibody response is delayed in acute HIV-1 infection (AHI): autologous neutralizing antibodies are not detectable until about 6 months after HIV-1 infection (1, 2), and broadly neutralizing antibodies only develop in a fraction of individuals over the course of several years of uncontrolled viremia (2–6). By contrast, neutralizing antibodies can be detected within 1–2 weeks of symptom onset for other viral infections or vaccination (7–10). HIV-1 Env has developed a number of mechanisms to evade the antibody response, including extensive glycosylation, conformational masking, escape mutations, and low expression on virions and virus-infected cells; however, early impaired immune activation during acute HIV-1 infection could also contribute to delayed antibody development (1, 11–15). Changes in lymph node structure and B-cell populations have been reported in early and chronic HIV-1 infection, but little is known about the germinal center (GC) response during the earliest stages of HIV-1 infection.

HIV-1 DNA can be detected in lymph nodes during the earliest stage of detectable HIV-1 in the blood, Stage 1 (S1), and reaches maximal levels as early as S2 (16). HIV-specific plasma antibodies are detected by immunoassay in S3 of AHI and by western blot thereafter. The first antibodies detected are gp41-specific antibodies followed by gp120-specific antibodies, but these initial antibodies do not contribute to viral control or the development of escape mutants during AHI (17). Analysis of antibody development in participants treated in different stages of AHI showed that participants who initiated antiretroviral therapy (ART) in S1 or S2 of AHI had limited development of gp120-specific antibodies, with lower levels of these antibodies than those who initiated treatment in S4/5 even after 24–48 weeks of ART (18). This analysis also suggested that only participants in S4/5 of AHI had more mature antibody development, with most participants having developed antibodies mediating antibody-dependent cellular cytotoxicity (ADCC) during AHI and all participants developing cross-clade ADCC activity after 1 year of ART (18). However, participants treated in these early acute time points do not develop autologous neutralizing antibodies in the absence of further antigen exposure through viral blips (19).

The stage-specific development of HIV-specific antibodies in AHI called for an analysis of the development of the GC response in lymph nodes. After activation, naïve B cells migrate to the periphery of the follicle where they interact with activated CD4^+^ T cells that have upregulated expression of CXCR5, a chemokine receptor that mediates trafficking toward the CXCL13-rich B-cell follicle (20–25). These interactions provide the signals necessary for the CD4^+^ T cells to differentiate into T follicular helper (Tfh) cells and the B cells to either differentiate into short-lived plasmablasts, which produce the first wave of antibodies, or to migrate to the GC for affinity maturation (26–28). Within the GC, Tfh cells provide B cells with survival signals that promote proliferation, somatic hypermutation, and selection to produce long-lived plasma cells and memory B cells with higher affinity receptors (24, 29, 30).

While Tfh cells express CXCR5 for trafficking to the CXCL13-rich follicle (23–25), they also express other chemokine receptors including CXCR3, which differentiates T helper 1 (Th1)-like Tfh cells from Th2/Th17-like Tfh cells (31). Frequencies of both CXCR3^−^ and CXCR3^+^ circulating Tfh (cTfh) cells have been associated with antibody development in response to viral infections and vaccinations (31–36), but appear to have different roles in the development of the humoral response. Studies of cTfh in the blood showed that CXCR3^−^ cTfh induced antibody production by naïve B cells better than CXCR3^+^ cTfh in co-culture (31). However, research in the mouse model suggests that CXCR3^+^ Tfh cells are involved in the early steps of GC formation (22, 37). Using fluorescently labeled cells, it has been shown that initial interactions between antigen-specific T and B cells during murine viral infections occur in the interfollicular zone where the CXCR3 ligands CXCL9 and CXCL10 are found (38–40), suggesting that CXCR3^+^ Tfh are involved in the initiation of the GC response (22, 37). Indeed, blocking CXCR3-mediated trafficking during influenza infection in mice resulted in an accumulation of influenza-specific CD4^+^ T cells in the T-cell zone and an impaired B-cell response (41). In the context of HIV infection, it was found that CXCR3^−^ cTfh from the blood of participants in AHI had an impaired capacity to induce antibody production by B cells *in vitro* (42). During chronic SIV infection of macaques, there was an increased frequency of CXCR3^+^ GC Tfh in lymph nodes and both CXCR3^+^ and CXCR3^−^ GC Tfh from these animals provided B cell help *in vitro* (43), but little is known about the role of CXCR3^+^ and CXCR3^-^ Tfh in lymph nodes during AHI.

Chronic HIV-1 infection (CHI) is associated with follicular hyperplasia marked with increased frequencies of both Tfh cells and GC B cells (44–46). There is also an accumulation of T-bet expressing B cells outside of GCs in lymph nodes during CHI (47). While expression of the transcription factor T-bet has been associated with exhausted B cells in chronic viral infections, studies have shown that frequencies of T-bet^+^ B cells increase in the blood after viral vaccination and AHI and suggest that these cells originate in the germinal center (48–50). Though CXCR3 was not measured on the T-bet^+^ B cells in all of these studies, its expression is known to be induced by T-bet in T and B cells (51, 52), and a recent in-depth analysis of peripheral B cells suggested that atypical T-bet^+^ B cells may arise after repeated antigenic stimulation of CXCR3^+^ B cells that compose an alternate lineage of memory B cells (48). Another study demonstrated the necessity of T-bet^+^ B cells in the GC response to viral infection in mice (53), but the role of these cells in human GCs has yet to be demonstrated.

Based on our previous analysis of antibody development in AHI in which participants in S4/5 of AHI had more mature antibody responses including increased ADCC (18), we hypothesized that significant GC development did not occur until after peak viremia in AHI. In this study, we sought to analyze how activation and differentiation of Tfh and B cells in the lymph nodes contribute to antibody responses during AHI and to determine predictors of long-term antibody production. Participants in the RV254 cohort in Thailand were diagnosed with HIV-1 during the different stages of AHI and immediately initiated ART (54). Some of these participants underwent optional inguinal lymphoid biopsies after diagnosis prior to ART initiation (55), providing a unique opportunity to perform cross-sectional analysis of lymph node mononuclear cells (LNMCs) during all stages of AHI and generate more informative and direct data about germinal center development than could be obtained from peripheral blood samples. We analyzed the phenotypes of B cells and Tfh cells in lymph nodes during AHI to identify which populations correlated with plasma antibody production both during AHI and after 1 year of ART.

## RESULTS

### Total GC B cell and Tfh cell frequencies are not associated with HIV-specific antibody levels in AHI

To study the early immune response in lymph nodes, we analyzed inguinal lymph node biopsies of participants in the RV254 and RV304 cohorts in Thailand. For participants in the RV254 cohort, biopsies were performed an average of 2 days after HIV-1 diagnosis in AHI and participants were staged according to a hybrid system that refines the identification of participants in S1 and S2 by detection of p24 using 4th generation immunoassays, with a median time to S1 of about 12.5 days after exposure (Table 1) (56, 57). Stages 1 and 2 are estimated to last about 5 days each, S3 encompasses the time of peak viral load (median 13 days after initial viral detection), and S4/5 includes the time of viral decline and establishment of setpoint viral load (56, 58). Samples from participants in RV304 with untreated chronic HIV-1 infection or healthy participants with no HIV-1 infection were also analyzed for comparison. Consistent with the predominance of male participants in these cohorts, all participants contributing to this analysis were male. HIV-1 DNA was detectable at low levels in LNMCs in S1 of AHI and increased significantly in S2 of AHI, as has been shown for this cohort (Fig. S1A) (16).

**TABLE 1:** Characteristics of participants included in this study

Characteristic	Value at stage of infection:						*P* value
	S1	S2	S3	S4/5	CHI	HIV^−^	
*N*	10	13	12	7	8	11	
Test results	PCR+/4G-/ 3G-/WB-	PCR+/4G+/ 3G-/WB-	PCR+/4G+/ 3G+/WB-	PCR+/4G+/ 3G+/WB+	N/A^*a*^	N/A^*a*^	
Age (years)	26	26	27	34	26	32	0.91^*c*^
Median (IQR)	(22-28)	(22-29)	(23-33)	(23-39)	(21-28)	(21-35)	
Sex (M:F)	10:0	13:0	12:0	7:0	8:0	12:0	>0.9999^*d*^
Log viral load	3.94		6.16	5.84	4.65	N/A^*a*^	<0.0001^*c*^
Median (IQR)	(3.68–4.52)	(5.33-6.23)	(5.69–6.87)	(5.44–6.82)	(4.14–5.19)		
CD4 count (cells/mL)	616	286	300	438	338	942	<0.0001^*c*^
Median (IQR)	(518-779)	(200-549)	(234-434)	(250-519)	(211-363)	(674–1144)	
HIV-1 subtype							
AE:AE/B:B	9:1:0	11:2:0	11:0:1	7:0:0	n.d.^*b*^	N/A^*a*^	0.64*^d^*

^*a*^ N/A, not applicable.

^*b*^ n.d., not determined.

^*c*^ *P* values from Kruskal-Wallis test.

^*d*^ *P* values from Fisher exact test.

**Fig 1:**
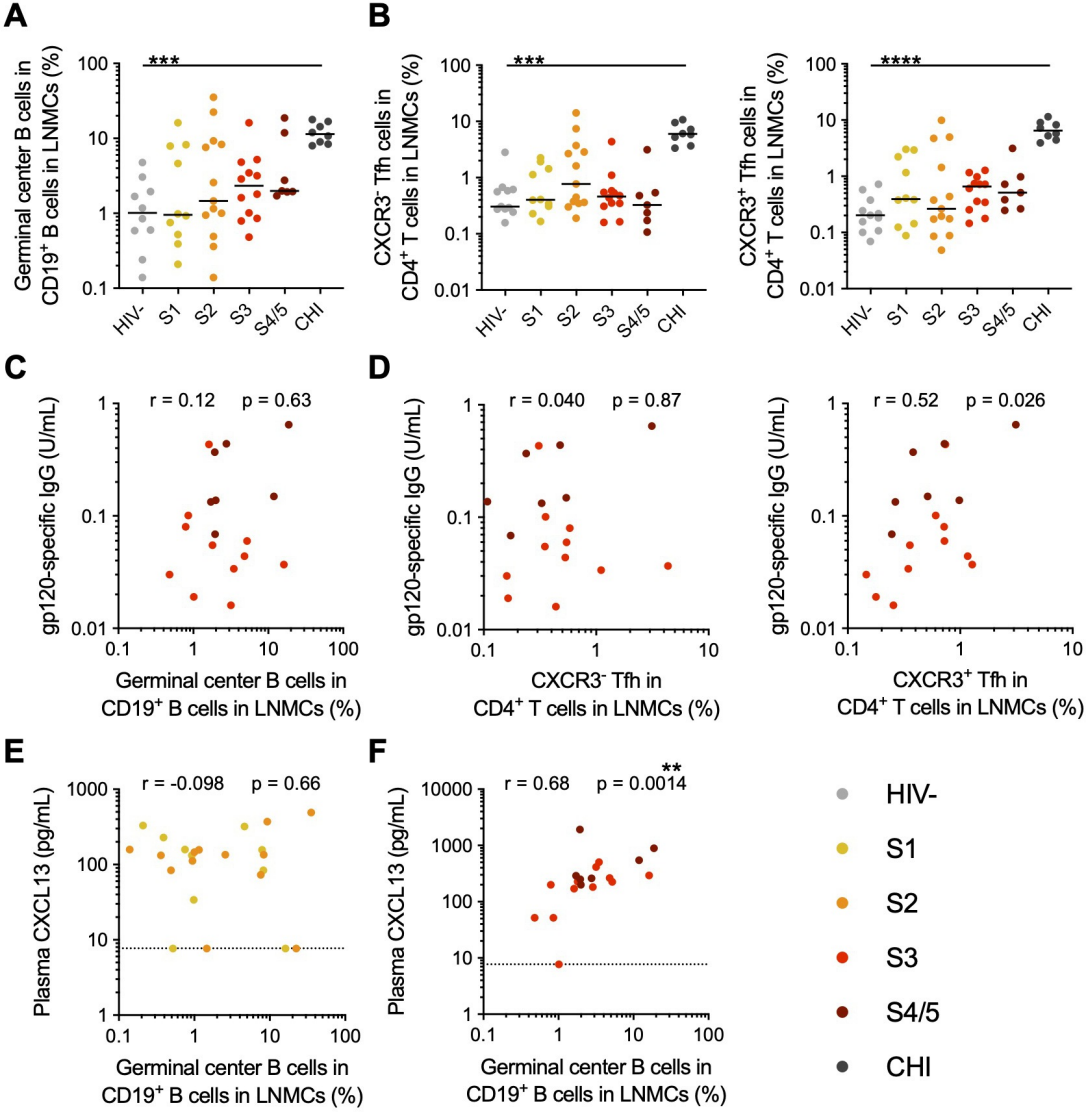
Germinal center B cell and T follicular helper cell frequencies do not increase in acute HIV-1 infection. (**A**) Frequencies of IgD^-^CD38^hi^ germinal center (GC) B cells were measured in LNMCs from participants prior to ART initiation in different stages of acute (AHI) or chronic (CHI) HIV infection by flow cytometry. GC B-cell frequencies were measured in individuals without HIV as a control. (**B**) Levels of gp120-specific IgG antibodies were measured in the plasma of participants in AHI, and the correlation with the frequency of GC B cells in lymph nodes in S3-5 of AHI is shown. (**C**) Frequencies of CXCR3^−^ and CXCR3^+^ Tfh (CXCR5^+^PD-1^hi^ CD4^+^ T cells) were measured in LNMCs of participants in the different stages of AHI or CHI by flow cytometry. (**D**) Correlations between the frequencies of CXCR3^−^ and CXCR3^+^ Tfh in LNMCs and plasma gp120-specific IgG antibody levels in S3-5 of AHI. CXCL13 levels were measured in plasma by Luminex, and correlations with GC B cell frequencies are shown for participants sampled in S1-2 (**E**) or S3-5 of AHI (**F**). Changes in cell populations during HIV-1 infection were measured by a Kruskal-Wallis test with Dunn’s multiple comparison to HIV^-^ controls. Correlations were measured with Spearman correlation. No correlations remained significant after correction for a false discovery rate (FDR) of 5%. *N* = 62 *P < 0.05, **P < 0.01, ***P < 0.001, ****P < 0.0001.

We previously reported that, although seroconversion can be identified at S3 of AHI, there is a significant increase in ADCC titer against CRF01_AE infected targets at S4/5 in this cohort (18). Given the increased HIV-specific IgG antibody levels and ADCC titer in S4/5 of AHI, we expected to find increased frequencies of GC B cells in lymph nodes during this time. However, we found no significant increase in the frequency of GC B cells (CD19^+^IgD^-^CD38^+^CD27^lo^CD20^hi^ B cells) during AHI, even at S4/5 when antibody levels and ADCC titer were elevated (Fig. 1A; Fig. S2A). In addition, there was no significant increase in Tfh cells (CXCR5^+^PD-1^hi^ CD4^+^ T cells) during AHI, whether looking at total Tfh or when gating on CXCR3^−^ and CXCR3^+^ Tfh (Fig. 1B; Fig. S2B and C). By contrast, we did measure significantly higher frequencies of GC B cells and Tfh cells in lymph nodes of participants with untreated CHI compared to HIV^-^ individuals (*P* < 0.001) as would be expected. cTfh populations are more often studied in human infection due to their accessibility in blood samples. We found there was a small, transient increase in the frequency of CXCR3^−^PD-1^+^, but not CXCR3^+^PD-1^+^, cTfh (CXCR5^+^ CD4^+^ T cells) in the blood in AHI, which reached significance in S2 (*P* < 0.01) but normalized by S4/5 compared to HIV^-^ participants (Fig. S2D and E). Frequencies of CXCR3^−^PD-1^+^ cTfh in the blood did correlate with frequencies of CXCR3^−^ Tfh in the lymph nodes during AHI (r = 0.64, *P* = 0.0031), but there was no correlation between the CXCR3^+^ populations in these compartments (Fig. S2F).

As HIV-specific antibody titers do not increase relative to HIV^−^ individuals until S3 of AHI (Fig. S1B), and gp41-specific antibodies can be cross-reactive to gut flora and derived from polyreactive memory B cells (59, 60), we focused on the production of gp120-specific antibodies in participants in S3 or later to identify correlations between GC function and *de novo* antibody responses in participants living with HIV-1. Still, we found that the frequency of GC B cells in lymph nodes did not correlate with the plasma levels of gp120-specific antibodies in S3-5 of AHI (Fig. 1C). It was only the frequency of CXCR3^+^ Tfh in the lymph nodes that correlated with plasma antibody levels during AHI (r = 0.52, *P* = 0.026), though this did not remain significant after correcting for false discovery rate (Fig. 1D; Fig. S2G and H). To further investigate whether there was increased GC activity during AHI, we measured plasma levels of CXCL13, the chemokine ligand of CXCR5, which has been shown to correlate with GC activity in lymph nodes (61). We found that there was a correlation between the GC B cell frequency in lymph nodes and plasma CXCL13 levels only when considering participants in S3–S5 of AHI (r = 0.68, *P* = 0.0014), but not for participants in S1–S2 of AHI (Fig. 1E and F). Together, these data suggest that despite a lack of measurable increase in Tfh and GC B cell frequency, there is GC activity occurring in lymph nodes during the later stages of AHI.

### CXCR3^+^ Tfh and B cells are activated in acute HIV-1 infection

**Fig 2:**
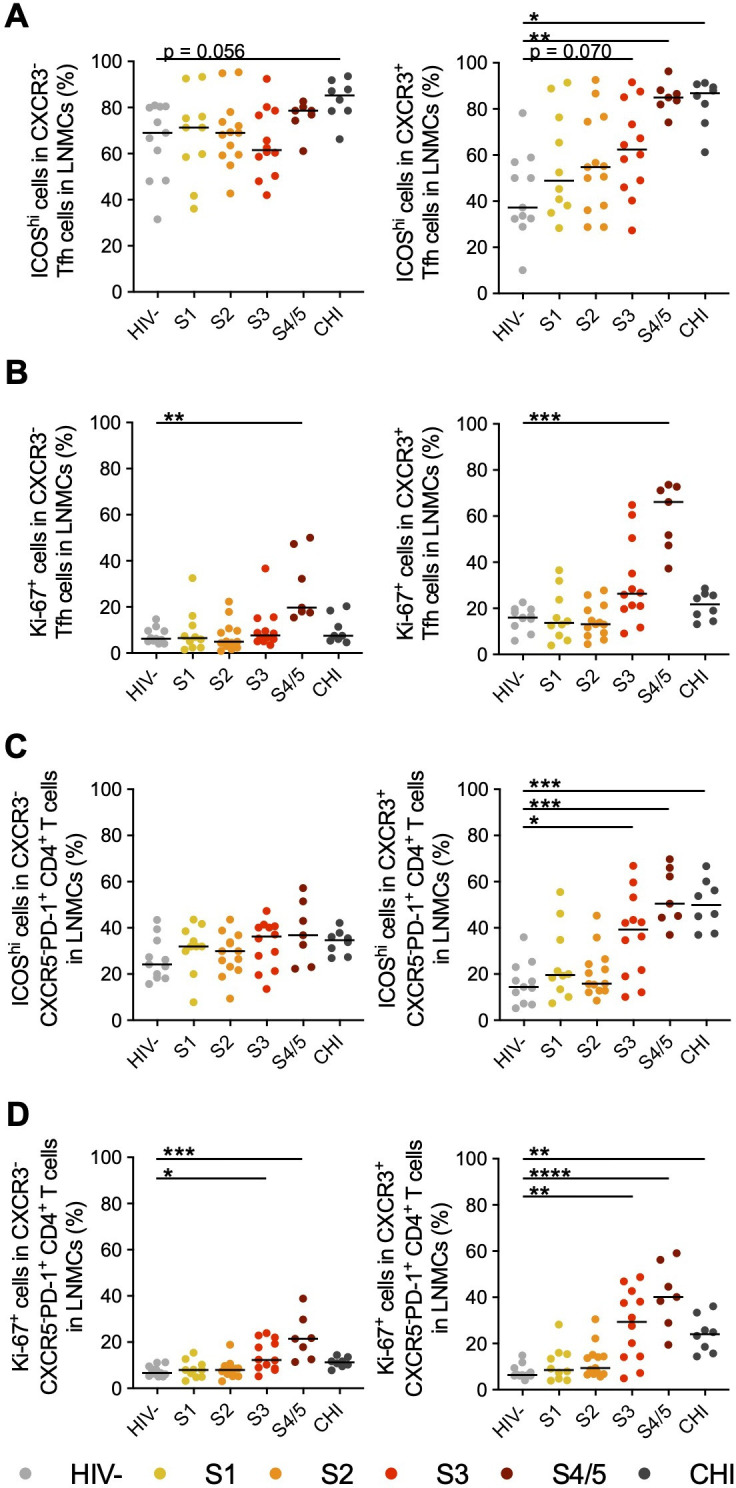
Increased frequencies of activated CXCR3^+^ Tfh cells during AHI. Frequencies of ICOS^hi^ cells (**A**) and Ki-67^+^ cells (**B**) within CXCR3^−^ and CXCR3^+^ Tfh populations were measured in LNMCs from participants prior to ART initiation in different stages of AHI or in CHI by flow cytometry. Frequencies of ICOS^hi^ cells (**C**) and Ki-67^+^ cells (**D**) within CXCR3^−^ and CXCR3^+^ CXCR5^-^PD-1^+^ non-Tfh CD4^+^ T-cell populations were measured by flow cytometry in LNMCs. Changes in frequencies of activated cells during HIV-1 infection were measured by a Kruskal-Wallis test with Dunn’s multiple comparison to HIV^−^ controls. *N* = 62 *P < 0.05, **P < 0.01, ***P < 0.001, ****P < 0.0001.

Proper Tfh differentiation is necessary for the initiation and maintenance of the GC reaction. ICOS signaling is required for Tfh differentiation and maintenance of the Tfh phenotype (62–67). The majority of CXCR3^−^ Tfh expressed high levels of ICOS in lymph node biopsies from all individuals regardless of their HIV status, with no significant increase in ICOS^hi^ CXCR3^−^ Tfh with AHI stage (Fig. 2A; Fig. S3A). By contrast, the frequency of ICOS^hi^ CXCR3^+^ Tfh increased steadily with disease stage, with significant differences in the frequency of these cells at S4/5 of AHI and CHI compared to HIV^-^ controls (*P* < 0.001 and *P* < 0.01, respectively) (Fig. 2A). To further elucidate their activation status, we measured Ki-67 expression to determine if there was increased proliferation of Tfh populations during AHI (Fig. S3B through D). Both CXCR3^–^ and CXCR3^+^ Tfh had increased frequencies of Ki-67^+^ cells at S4/5 of AHI compared to HIV^-^ controls (*P* < 0.01 and *P* < 0.001, respectively), but CXCR3^+^ Tfh had higher frequencies of Ki-67^+^ cells than CXCR3^−^ Tfh (median 66% vs 20%, respectively) (Fig. 2B). Similar increases in proliferating cells were measured within the GC Tfh (CXCR5^hi^PD-1^hi^) populations, though the frequencies of these populations within CD4^+^ T cells were extremely low (data not shown). This activation of Tfh populations was slightly delayed compared to non-Tfh cells (CXCR5^−^ CD4^+^ T cells), for which the majority of activated cells were found within the PD-1^+^ population. Increased frequencies of ICOS^hi^ and Ki-67^+^ cells could be measured at S3 of AHI within the CXCR3^+^ (*P* < 0.05 and *P* < 0.01, respectively) and CXCR3^−^ (Ki-67: *P* < 0.05) populations of CXCR5^-^PD-1^+^ CD4^+^ T cells (Fig. 2C and D).

Both CXCR3^−^PD-1^+^ and CXCR3^+^PD-1^+^ cTfh in the blood also showed signs of activation during AHI, with CXCR3^−^ cells having elevated frequencies of ICOS^hi^ (*P* < 0.05) and Ki-67^+^ (*P* = 0.061) cells at S4/5 compared to HIV^−^ individuals, and CXCR3^+^ cells having significant increases starting at S3 (ICOS^hi^: *P* < 0.05; Ki-67^+^: *P* = 0.064) (Fig. S3E and F). The frequencies of activated CXCR3^+^PD-1^+^ cTfh in the blood moderately correlated with the frequencies of activated CXCR3^+^ Tfh in the lymph nodes (ICOS^hi^: r = 0.41, *P* = 0.078; Ki-67^+^: r = 0.56, *P* = 0.013)(Fig. S3G), but the frequencies of activated CXCR3^−^PD-1^+^ cTfh in the blood did not correlate with activated CXCR3^−^ Tfh in the lymph nodes. Instead, frequencies of CXCR3^−^PD-1^+^ cTfh correlated with the frequencies of activated CXCR3^+^ Tfh in the lymph nodes (ICOS^hi^: r = 0.55, *P* = 0.016; Ki-67^+^: r = 0.54, *P* = 0.017)(Fig. S3H). These data suggest that while measurement of CXCR3^+^ cTfh activation in the blood may give some indication of CXCR3^+^ Tfh activation in the lymph nodes, cTfh populations in the blood do not directly correlate with Tfh populations in the lymph nodes and analysis of LNMCs is necessary to understand the development of humoral responses in AHI.

**Fig 3:**
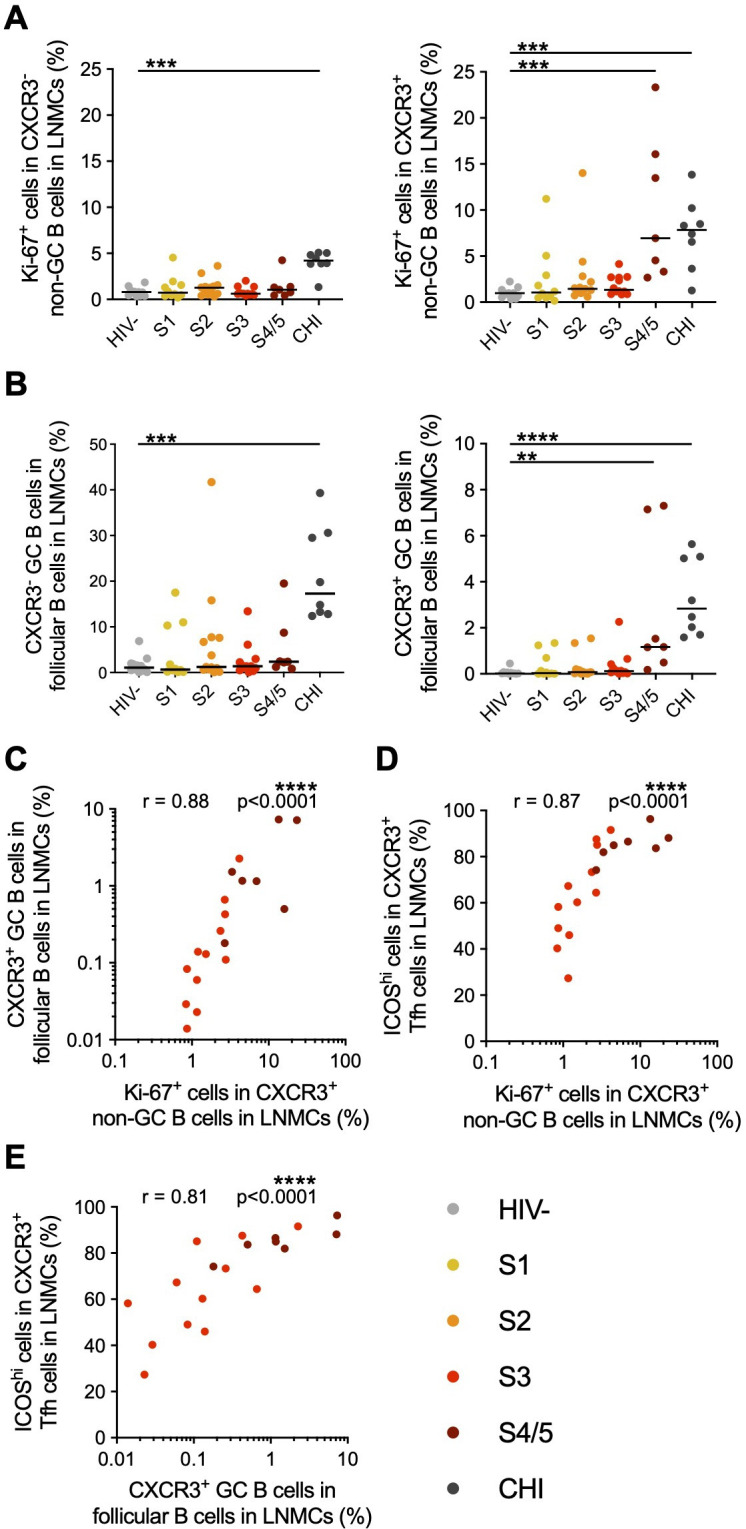
CXCR3^+^ B cells are activated in lymph nodes during AHI. (**A**) The frequency of Ki-67^+^ cells within CXCR3^−^ and CXCR3^+^ follicular non-GC B cells (CXCR5^+^Bcl-6^−^) was measured by flow cytometry in LNMCs from participants prior to ART initiation in different stages of AHI or CHI. (**B**) The frequencies of CXCR3^−^ and CXCR3^+^ GC B cells (CXCR5^+^Bcl-6^+^) in LNMCs were measured by flow cytometry. (**C**) Correlation between the frequency of Ki-67^+^ cells within CXCR3^+^ non-GC B cells and the frequency of CXCR3^+^ GC B cells. Correlations were also measured between the frequency of Ki-67^+^ cells within CXCR3^+^ non-GC B cells (**D**) or CXCR3^+^ GC B cells (**E**) and the frequency of ICOS^hi^ CXCR3^+^ Tfh during S3-5 of AHI. Changes in frequencies of cell populations during HIV-1 infection were measured by a Kruskal-Wallis test with Dunn’s multiple comparison to HIV^−^ controls. Correlations were measured by Spearman correlation. All correlations remained significant after correction for an FDR of 5%. *N* = 62 *P < 0.05, **P < 0.01, ***P < 0.001, ****P < 0.0001.

As early activation of Tfh cells occurs through interactions with B cells at the T-B border, we sought to identify B cells that were also activated by these interactions. After receiving stimulating signals at the T-B border, B cells undergo proliferation before entering the germinal center (68); therefore, we measured the frequency of proliferating non-GC B cells in the follicle during AHI. An anti-CXCR3 antibody was not included in the flow cytometry panel for B-cell phenotyping; thus, we identified B cells within the CD4^+^ T-cell phenotyping panel (Table S1). Follicular B cells were identified as CXCR5^+^ cells within the CD3^−^CD4^−^CD8^−^ population (Fig. S4A), and CD19^+^ expression was further confirmed in these cells (Fig. S4B). Bcl-6 expression was used to differentiate non-GC (bcl-6^−^) and GC (bcl-6^+^) B cells, with the frequency of GC B cells identified by bcl-6 expression correlating strongly with the frequency of GC B cells identified by CD38, IgD, CD27, and CD20 expression (Fig. S2A, S4A, and C). Measuring Ki-67^+^ expression on non-GC B cells in the lymph nodes, we found that there was increased proliferation specifically within the CXCR3^+^ population in S4/5 in AHI (*P* < 0.001), whereas both populations of non-GC B cells had increased proliferation in CHI (Fig. 3A). Furthermore, though the frequency of GC B cells was low, when we gated GC B cells based on CXCR3 expression, there was a significant increase in CXCR3^+^ GC B cells in lymph nodes in S4/5 of AHI (*P* < 0.01), whereas frequencies of both CXCR3^−^ and CXCR3^+^ GC B cells were elevated in CHI (*P* < 0.001 and *P* < 0.0001, respectively)(Fig. 3B). This increase in CXCR3^+^ GC B cells strongly correlated with the frequency of Ki-67^+^ CXCR3^+^ non-GC B cells (r = 0.88, *P* < 0.0001)(Fig. 3C), and these B-cell populations also correlated strongly with CXCR3^+^ Tfh activation (r = 0.87 and r = 0.81, respectively, *P* < 0.0001)(Fig. 3D and E) and proliferation (r = 0.74, *P* = 0.0003 and r = 0.56, *P* = 0.012, respectively)(Fig. S4D and E). A matrix showing all correlations between Tfh and B cells is provided in Fig. S5.

To determine whether antigen burden affected the activation of Tfh and B cells in the different stages of AHI, we analyzed correlations with viral measures. As viral load peaks during S3 and then decreases to set-point in S5, we used an imputed viral load area under the curve (AUC) which was modeled on data from the RV217 untreated acute infection cohort and accounts for the level of viremia before diagnosis to provide an estimate of total antigen burden (69). There were no significant correlations between the frequencies of CXCR3^−^ or CXCR3^+^ proliferating Tfh cells, proliferating non-GC B cells, or GC B cells and the imputed viral load AUC prior to ART initiation (data not shown). There were also no correlations between Tfh and B-cell populations with the levels of total HIV-1 DNA or integrated HIV-1 DNA measured in CD4^+^ LNMCs (Fig. S6). Together, these data suggest that CXCR3^+^ Tfh and CXCR3^+^ B cells are activated first in AHI to initiate the GC response, and although significant levels of activation did not occur until after peak viremia, they were not associated with viral burden.

### Proliferating CXCR3^+^ Tfh cells in acute HIV-1 infection correlate with short- and long-term antibody levels

**Fig 4:**
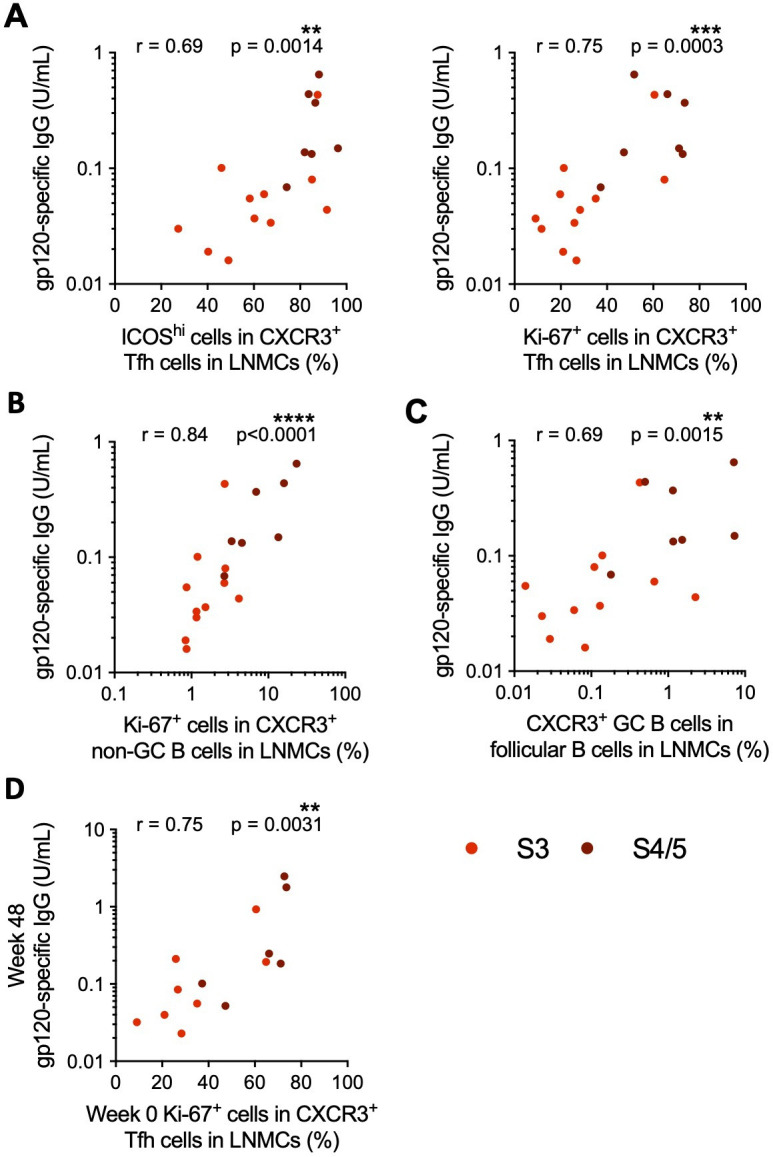
The frequencies of proliferating CXCR3^+^ Tfh and CXCR3^+^ GC B cells in lymph nodes correlated with plasma gp120-specific antibody levels in AHI. Correlations between plasma gp120-specific antibody levels at the time of diagnosis and the frequency of ICOS^hi^ or Ki-67^+^ cells in CXCR3^+^ Tfh (**A**), Ki-67^+^ cells in CXCR3^+^ non-GC B cells (**B**), and CXCR3^+^ GC B cells (**C**) in lymph nodes prior in S3-5 of AHI. (**D**) Levels of gp120-specific antibodies were measured in the plasma by ELISA after 48 weeks of ART. The correlation between the frequency of Ki-67^+^ cells within CXCR3^+^ Tfh cells in lymph nodes prior to ART initiation in AHI and gp120-specific antibody levels after 48 weeks of ART is shown. All correlations were measured by Spearman correlation. Correlations that remained significant after correction for an FDR of 5% are indicated by asterisks (*). *N* = 18 for *A-C*; *N* = 14 for *D*.

To determine what cell populations might support antibody production, we measured correlations between the Tfh and B-cell populations and Env-specific antibody levels during AHI and after ART. The frequencies of both ICOS^hi^ and Ki-67^+^ cells within CXCR3^+^PD-1^+^ cTfh correlated with gp120-specific antibody levels during AHI; however, these correlations did not hold after correction for false discovery rate (Fig. S7A and B). The frequencies of ICOS^hi^ and Ki-67^+^ cells in CXCR3^+^ Tfh cells in lymph nodes correlated with gp120-specific antibody levels (r = 0.69, *P* = 0.0014; r = 0.75, *P* = 0.0003, respectively)(Fig. 4A). By contrast, there were only weak trends in association between the frequencies of ICOS^hi^ and Ki-67^+^ CXCR3^−^ Tfh cells with plasma gp120-specific IgG antibody levels (Fig. S7C). Within B cells, the frequencies of both the proliferating CXCR3^+^ non-GC B cells and total CXCR3^+^ GC B cells correlated with gp120-specific antibody levels (r = 0.84, *P* < 0.0001 and r = 0.69, *P* = 0.0015, respectively), but there were no correlations between CXCR3^−^ B-cell populations and antibody levels (Fig. 4B and C; Fig. S7D and E). These data indicate that activation of CXCR3^+^ Tfh and B-cell populations in the lymph nodes are associated with Env-specific antibody production in AHI.

To understand what Tfh and B-cell populations present in lymph nodes during AHI are important for long-term HIV-specific antibody development, we calculated correlations with antibody levels after ART. After 48 weeks of ART, when all participants had an undetectable viral load (<20 copies/mL), most of the correlations between antibody levels and the frequencies of Tfh and B-cell populations at AHI were lost. Neither the frequency of Ki-67^+^ CXCR3^+^ non-GC B cells nor CXCR3^+^ GC B cells in lymph nodes in AHI significantly correlated with antibody levels after 48 weeks of ART (Fig. S8A and B). Among the Tfh populations, only the frequency of proliferating Ki-67^+^ CXCR3^+^ Tfh in the lymph nodes in AHI correlated with gp120-specific IgG levels at 48 weeks of ART (r = 0.75, *P* = 0.0031)(Fig. 4D; Fig. S8C and D). Together, these data suggest that activation and proliferation of CXCR3^+^ Tfh and B cells in the lymph nodes during AHI are associated with the development of the *de novo* gp120-specific antibody response, and Ki-67^+^ CXCR3^+^ Tfh in the lymph nodes in AHI specifically correlated with long-term gp120-specific antibody levels.

**Fig 5:**
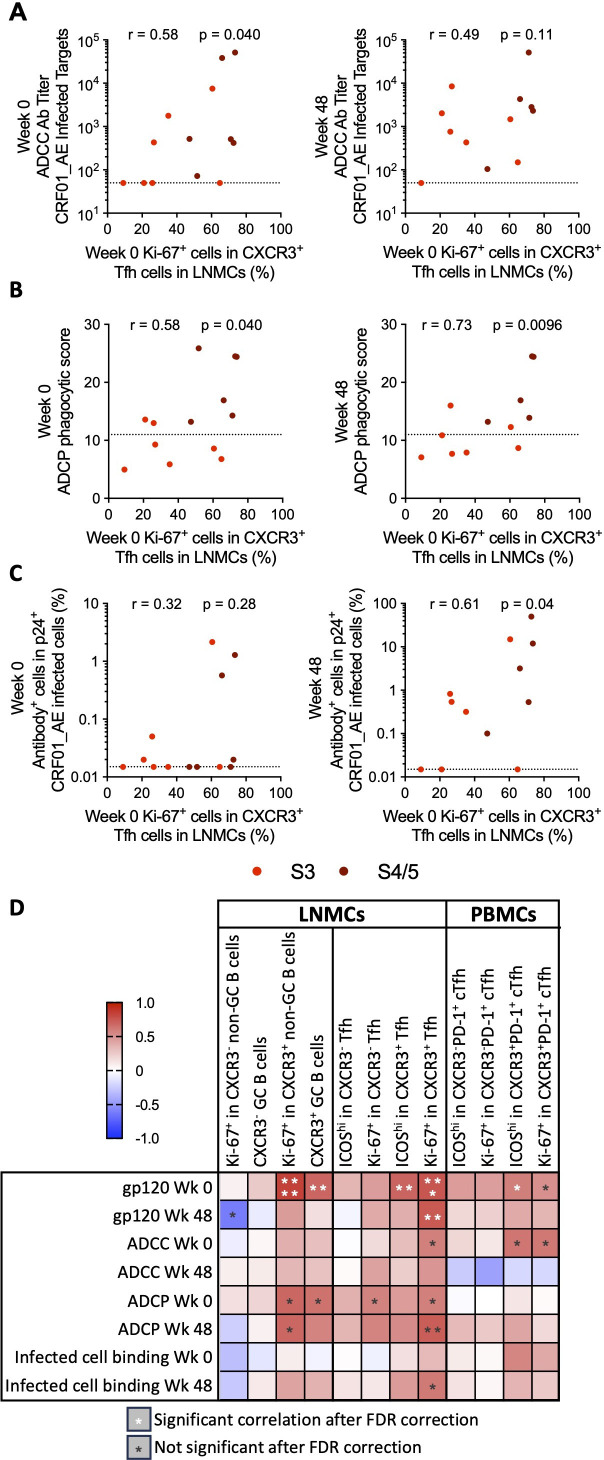
The frequencies of proliferating CXCR3^+^ Tfh cells in lymph nodes correlated with increased antibody binding. Correlations between Ki-67^+^ CXCR3^+^ Tfh cells in lymph nodes at the time of diagnosis and the ADCC antibody titer (**A**) and ADCP phagocytic score (**B**) measured in plasma collected at ART initiation in S3–5 of AHI and after 48 weeks of ART. (**C**) Correlation between Ki-67^+^ CXCR3^+^ Tfh cells in lymph nodes at the time of diagnosis and the frequency of p24^+^ CRF01_AE infected cells bound by plasma antibody from week 0 or week 48. All correlations were measured by Spearman correlation. *N* = 13 for week 0; *N* = 12 for week 48. (D) A heatmap shows the Spearman *r* values for correlations between Tfh and B cell populations at the time of diagnosis and antibody measures at diagnosis or after 48 weeks of ART for participants who initiated treatment in S3-5 of AHI. All correlations were measured by Spearman correlation. Correlations that remained significant after correction for a FDR of 5% are indicated by white asterisks (*). *P < 0.05, **P < 0.01, ***P < 0.001, ****P < 0.0001.

Antibody functionality, rather than titer, has been associated with vaccine protection and viral control after treatment interruption (70–72). To determine whether the development of ADCC activity was also associated with the activation of lymph node populations, we evaluated correlations between ADCC titers at ART initiation and after 48 weeks of ART with Tfh and B-cell populations in the lymph nodes of a portion of participants who initiated ART in S3–S5 of AHI. We found that only the frequency of Ki-67^+^ CXCR3^+^ Tfh in lymph nodes correlated with ADCC titer at the time of ART initiation (r = 0.58, *P* = 0.040), while only a trend in correlation remained after 48 weeks of ART (Fig. 5A; Fig. S9). Furthermore, we found that, though low, antibody-dependent cellular phagocytosis (ADCP) scores correlated with the frequencies of proliferating CXCR3^−^ (r = 0.57, *P* = 0.04) and CXCR3^+^ (r = 0.58, *P* = 0.04) Tfh, proliferating CXCR3^+^ non-GC B cells (r = 0.69, *P* = 0.01), and total CXCR3^+^ GC B cells (r = 0.64, *P* = 0.02) in lymph nodes at the time of ART initiation. However, ADCP phagocytic scores after 48 weeks of ART only remained significantly correlated with frequencies of proliferating CXCR3^+^ Tfh (r = 0.73, *P* = 0.0096) and CXCR3^+^ non-GC B cells (r = 0.68, *P* = 0.01)(Fig. 5B; Fig. S10).

Although early initiation of ART precluded the development of neutralizing antibodies, it has been shown that individuals who went on to develop broadly neutralizing antibodies had increased antibody binding to trimeric Env as early as 1 month post-HIV-1 acquisition compared to those who were non-broad neutralizers (73). Antibody binding to HIV-infected cells was quite low in AHI (18), and there were no correlations with the frequencies of Tfh or B-cell populations in the lymph nodes at the time of ART initiation (Fig. 5C; Fig. S11). However, the frequency of Ki-67^+^ CXCR3^+^ Tfh in the lymph nodes in AHI correlated with increased antibody binding to HIV-infected cells after 48 weeks of ART . A matrix summarizing antibody correlations with Tfh and B-cell populations is provided in Fig. 5D. Together, these data suggest that CXCR3^+^ Tfh proliferation in the lymph node during AHI is associated with long-term HIV-specific antibody levels, increased antibody binding to infected cells over time, and development of Fc receptor-mediated antibody effector function.

### A higher frequency of CXCR3^+^ Tfh is infected with HIV in acute HIV-1 infection

**Fig 6:**
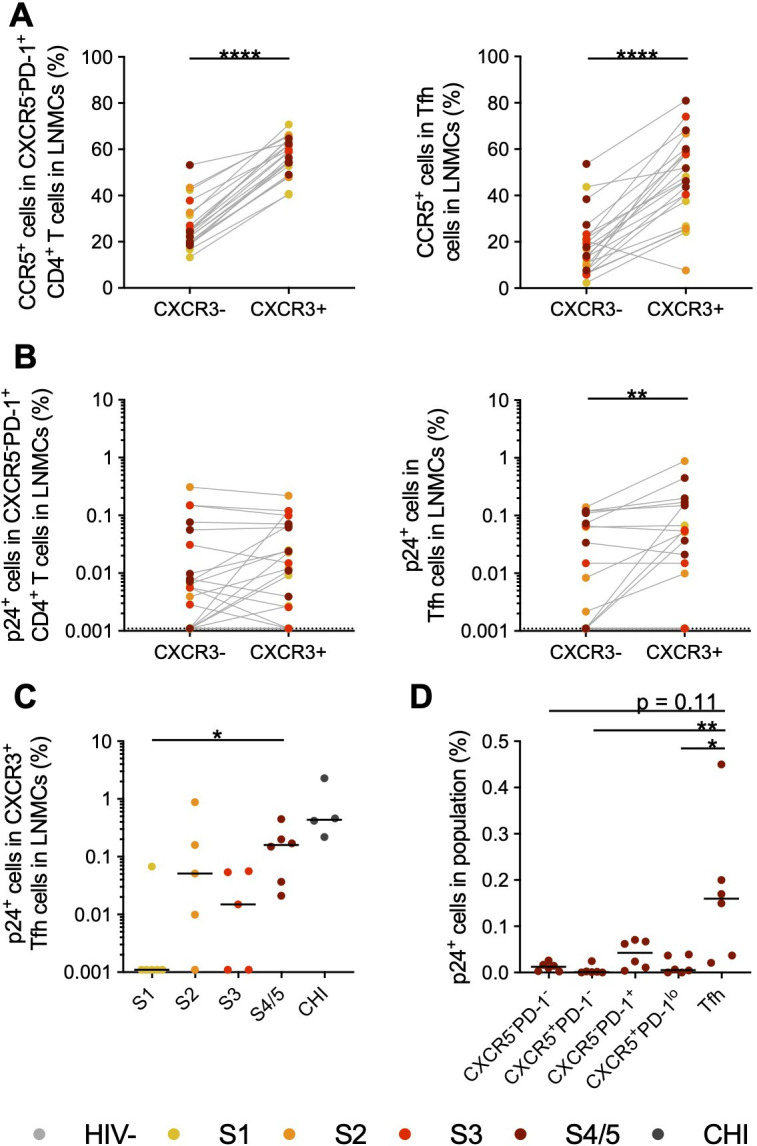
Increased permissiveness and infection of CXCR3^+^ Tfh cells in lymph nodes. (**A**) The frequency of CCR5^+^ cells within CXCR3^−^ and CXCR3^+^ CXCR5^-^PD-1^+^ non-Tfh or Tfh populations was measured in LNMCs from participants at different stages of AHI. (**B**) The frequency of HIV-1 p24-producing cells was measured by HIV-Flow in LNMCs from participants in AHI. Comparisons of the frequency of p24^+^ cells in CXCR3^−^ and CXCR3^+^ populations are shown for CXCR5^-^PD-1^+^ non-Tfh and Tfh cells. (**C**) Frequencies of p24^+^ cells within CXCR3^+^ Tfh cells in LNMCs from participants in AHI and CHI. (**D**) Frequencies of p24^+^ cells within different CXCR3^+^ memory CD4^+^ T-cell populations in LNMCs from participants in S4/5 of AHI. Differences in the frequency of CCR5^+^ or p24^+^ cells between CXCR3^−^ and CXCR3^+^ cell populations were measured by a Wilcoxon test. Changes in frequencies of p24^+^ cells between different stages of AHI were measured by a Kruskal-Wallis test with Dunn’s multiple comparison between stages. Changes in frequencies of p24^+^ cells between different CXCR3^+^ CD4^+^ T-cell populations in S4/5 were measured by a Kruskal-Wallis test with Dunn’s multiple comparison to Tfh cells. *N* = 26 *P < 0.05, **P < 0.01, ****P < 0.0001.

These data show that activation of CXCR3^+^ Tfh was associated with HIV-specific antibody levels in AHI and after ART; however, CXCR3^+^ Tfh activation was not significantly elevated until after peak viremia. CD4^+^ follicular regulatory T (Treg) cells have been shown to affect Tfh/B-cell interactions in CHI (74), but we did not find any changes in CD4^+^ follicular Treg frequencies in AHI (Fig. S12A), suggesting that these cells may not be affecting early Tfh activity. We did find that there was a trend toward more CD1c^+^ myeloid dendritic cells (mDCs) that expressed DC-SIGN in the lymph nodes in S2 of AHI (*P* = 0.11) (Fig. S12B), suggesting an increased potential for trans-infection of HIV-specific CD4^+^ T cells in S2 of AHI (75). It has been shown that HIV-1 efficiently infects CCR5^+^PD-1^+^ CD4^+^ T cells from tonsil biopsies of HIV^-^ participants *in vitro* (76), so we investigated whether there were differences in CCR5 expression between CXCR3^−^ and CXCR3^+^ cells in AHI. Indeed, CXCR3^+^ cells more frequently expressed CCR5 than CXCR3^−^ cells both within the Tfh and CXCR5^-^PD-1^+^ non-Tfh CD4^+^ T-cell populations (*P* < 0.0001) (Fig. 6A; Fig. S12C). However, when we measured p24^+^ cells in lymph nodes by HIV-Flow (Fig. S12D), we found that only the Tfh had increased frequencies of infected CXCR3^+^ cells compared to CXCR3^−^ cells during AHI (*P* < 0.01)(Fig. 6B; Fig. S12E), but we did not find a significant difference in the frequency of p24^+^ cells expressing CCR5 in these subsets (Fig. S12F). Interestingly, the majority of participants had p24^+^ CXCR3^+^ Tfh in the lymph nodes as early as S2 of AHI, and all participants in S4/5 of AHI had detectable p24^+^ CXCR3^+^ Tfh (Fig. 6C), a pattern similar to that seen for CXCR3^+^ CXCR5^-^PD-1^+^ non-Tfh cells (Fig. S12G). Although Tfh makes only a small contribution to the total pool of p24^+^ cells during AHI due to their low frequency (Fig. 1B)(77), CXCR3^+^ Tfh had the highest frequency of p24^+^ cells by S4/5 of AHI (median 0.16%) (Fig. 6D), a change from S3 when there were no differences in infection frequency between populations (Fig. S12H). These data suggest that S4-5 may be a critical transition period when HIV-1-producing cells are established in the B-cell follicles.

## DISCUSSION

Through access to unique lymph node biopsies and analysis of LNMCs from individuals diagnosed in different stages of AHI, we show here that activation of CXCR3^+^ Tfh and B cells is associated with antibody development in AHI and after long-term ART, suggesting a potential role for these cells in the induction of the GC response against HIV-1. Here we showed that CXCR3^+^ Tfh were significantly activated starting in S3, as measured by expression of Ki-67 and high levels of ICOS, which is necessary for Tfh differentiation (63, 78). These data are consistent with observations in murine infection studies that found CXCR3^+^ Tfh are involved in the early steps of GC formation (22, 37) and that early IL-21-producing Tfh also express *Ifng*, a cytokine produced by CXCR3^+^ Tfh (43, 79). While sample availability limited the analysis of Tfh function and location in the current study, these data provide evidence that CXCR3^+^ Tfh cells are activated first in AHI, suggesting a similarly important role for these cells in acute viral infection in humans as has been shown in murine and macaque models.

Whereas CXCR3 expression by T cells is important for T cell positioning at the T-B border and initiation of the GC response, its expression in B cells is important for the quality of the GC response by promoting B-cell trafficking to the dark zone (52). CXCR3 expression itself is not required for the development of GC B cells (41) but it correlates strongly with T-bet expression in GC B cells (51, 52), which is induced by IFNγ produced by CXCR3^+^ Tfh and is associated with antibody class switching and increased somatic hypermutation (41, 52, 80). We previously reported that individuals who initiated treatment in S4/5 of AHI had better antibody development, including increased cross-clade antibody responses after suppressive ART (18), and have shown here that it is in S4/5 when increased frequencies of CXCR3^+^ GC B cells can be found in the LN. Unfortunately, despite this GC activity individuals who initiated ART in acute HIV infection only developed autologous neutralizing antibodies if they experienced further viremia after ART (19). Further studies are needed to understand how the GC response progresses after ART initiation and what factors limit development of neutralizing antibodies in these participants.

As blood samples are more readily available for analysis, many studies have focused on cTfh as a surrogate of Tfh in lymph nodes, but the association between the frequency and function of cTfh and Tfh is still not well understood. We found that frequencies of activated CXCR3^+^ Tfh in the lymph nodes during AHI correlated with activated cTfh in the blood, regardless of CXCR3 expression. Our data suggest that both CXCR3^−^ and CXCR3^+^ cTfh may be progeny of the activated CXCR3^+^ Tfh during AHI, an observation that is in line with a recent study which showed that both CXCR3^−^ and CXCR3^+^ cTfh have shared clonotypes with CXCR3^+^ tonsillar Tfh (81), and may help explain disparate findings of correlations between neutralizing antibody production and CXCR3^−^ versus CXCR3^+^ cTfh frequencies during HIV-1 infection (82, 83). Further studies are necessary to determine whether both CXCR3^−^ and CXCR3^+^ cTfh arise from the same Tfh population, if correlations between total cTfh and Tfh populations exist later in infection, and if these observations are specific to AHI.

As there are, to our knowledge, no other studies that have analyzed Tfh and GC B-cell responses in the earliest stages of acute viral infections in humans, no data exist on the timing of these responses in humans. It is intriguing to consider that the lack of significant Tfh and follicular B-cell proliferation until S4/5 of AHI may represent delayed differentiation of these cells, which, in turn, contributes to the delayed development of autologous neutralizing antibodies compared to other infections. We showed here that significant increases in CXCR3^+^ Tfh and B-cell activation did not occur in the lymph nodes until S4/5 of AHI, corresponding to more than 3 weeks after infection (84), a time when neutralizing antibodies can already be detected in the plasma in other infections and vaccinations (7–10). For instance, analysis of fine-needle aspirates of draining lymph nodes after SARS-CoV-2 vaccination showed that ~10-20% of GC B cells bound to spike protein by 3 weeks after primary vaccination (85), a time at which the majority of participants already had low but detectable neutralizing antibody titers (10).

Tfh activation in the lymph nodes during AHI could be inhibited by a number of factors. Regulatory cells, including follicular CD4^+^ and CD8^+^ Treg cells and a recently described population of follicular regulatory innate lymphoid cells, have all been found to impair Tfh/B-cell interactions in CHI (74, 86, 87). We found no changes in the frequencies of follicular CD4^+^ Tregs in lymph nodes during AHI, but further analysis is needed to determine if other regulatory populations are elevated in AHI. Tfh cell activation could alternatively be affected by elevated cytokine production in AHI. TNFα levels rise and fall in the plasma during AHI coincident with viral load (88), and TNFα has been shown to inhibit GC formation in other bacterial and viral infections (89, 90). IFNα levels are also elevated in the blood during AHI, peaking before viral load (88), and may alter dendritic cell priming of CD4^+^ T cells to promote differentiation into Th1 rather than Tfh cells (91).

Early infection of Tfh precursors by HIV-1 could also affect our detection of Tfh activation and subsequent GC development in AHI. We found that mDCs tended to have increased the expression of DC-SIGN in S2 of AHI, increasing their potential to cause trans-infection of HIV-specific CD4^+^ T cells (92). In turn, early infection of HIV-specific CD4^+^ T cells could lead to their depletion by cytopathic effect or through elimination by cytotoxic NK cells or CD8^+^ T cells, especially during the early stages of differentiation before they enter the follicle, preventing their differentiation into Tfh cells and affecting our ability to accurately measure viral infection in these cells. Alternatively, HIV infection of Tfh precursor cells could modulate the differentiation signals they receive. In particular, Nef has been shown to impair immunologic synapse formation and modulate T-cell receptor signaling (93, 94), and productively infected CD4^+^ T cells in lymph nodes during AHI express low levels of CD4 (77), presumably due to downregulation by Nef and Vpu (95).

Tfh cells have been identified as a major cellular reservoir for HIV-1 in CHI and after ART (45, 96), but only a small portion of p24^+^ cells have a Tfh phenotype during AHI (77). Despite contributing minimally to the overall pool of HIV-infected cells, CXCR3^+^ Tfh had the highest rate of infection by S4/5 of AHI, when they also had a significant increase in proliferation. This is important because the increase of antibody production in S4/5 of AHI would facilitate the deposition of HIV-1 immune complexes on the FDC network in the follicles (97), and HIV-1 bound to FDCs is highly infectious to CD4^+^ T cells (98). Indeed, it has been shown that 1–2 months after seroconversion, during S5 of AHI, there is weak staining of HIV-1 on the FDC network (97). The increased activation and infection of CXCR3^+^ Tfh in S4/5 of AHI together with increased virus deposition on the FDC may contribute to seeding of HIV-1-infected cells in the follicle at this time. To this point, it has recently been shown that participants who initiated treatment in S3–S5 of AHI had higher levels of viral RNA^+^ cells in the follicle during ART (99), and that CXCR3^+^ Tfh cells harbored higher levels of HIV-1 RNA after ART (100). These data suggest that S4/5 of AHI may be a critical transition point when CXCR3^+^ Tfh establish the HIV-1 reservoir in the follicle and these results should be taken into consideration when administering therapeutic interventions at the time of HIV-1 diagnosis and ART initiation in AHI.

We provide here the first analysis of GC development in human lymph nodes in the earliest stages of AHI. This was a cross-sectional study, but the relative uniformity of the study population in regards to gender, HIV-1 clade, and residence in Thailand allowed us to analyze the developing immune response in lymph nodes with minimal variance. Still, studies need to be performed to determine whether similar results are found in cohorts that include women, people infected with different HIV-1 clades, and people from other regions of the world. Furthermore, we were limited to the study of Tfh and B-cell activation in inguinal lymph node biopsies, but the timing and degree of activation may be different in other lymphoid tissues. We were unable to measure HIV-specific responses in these samples due to the low cell number and early infection stage, but HIV-specific Tfh and B cells may be measured in future studies of lymph node biopsies from participants in S4-5 of AHI, as we have shown here that there are significant increases in proliferation of CXCR3^+^ Tfh and B cells in these stages. The current study was also limited by a lack of access to tissue blocks, which would have allowed us to measure changes in absolute numbers of cells rather than relative frequencies, and to perform histological examination of GC formation. While we showed that CXCR3^+^ Tfh and B cells are activated in AHI and could play an important role in HIV-specific antibody development, it is not clear whether a similar role of CXCR3^+^ Tfh and B cells would occur in infections that do not induce a strong Th1 response. Furthermore, it is not clear whether CXCR3^+^ Tfh and B cells play a similarly important role in the early stages of a memory response, as would occur during HIV rebound after treatment interruption.

Further studies are needed to elucidate the mechanisms that regulate CXCR3^+^ Tfh activation and subsequent GC activity during AHI and after early ART initiation. It has been shown that autologous neutralizing antibodies block viral outgrowth from the HIV-1 reservoir (101), but it takes months for neutralizing antibodies to develop after HIV-1 infection and individuals who initiate ART in AHI do not develop autologous neutralizing antibody titers in the absence of viral blips (1, 2, 18, 19). It is possible that the key activities that drive the development of autologous neutralizing antibodies have not yet occurred in the lymph nodes in S1-S5 of AHI; however, increased binding of B cells to the founder virus in AHI has been associated with the eventual development of broad neutralization (73), suggesting that early virus B-cell interactions are important for the development of neutralizing antibodies. Antibody maturation during HIV-1 infection also relies on co-evolution with the virus, with viral diversification around the binding site driving affinity maturation of the antibody and vice versa (102). Thus, the decreased antigen burden and lower viral diversity that result from early initiation of ART may preclude the development of neutralizing antibodies, even if the key activities in the lymph node have already begun. Understanding the earliest pathogenic mechanisms in the lymph nodes that limit antibody development after early ART initiation may facilitate the development of more effective interventions to target the immune responses in the lymph nodes. However, the benefits of increasing antibody development must be weighed against the potential of increasing HIV-1 reservoir seeding in the lymph node upon targeted Tfh activation during AHI.

## MATERIALS AND METHODS

### Experimental design

Peripheral blood and lymph node biopsies were analyzed to determine impairments in GC development during acute HIV-1 infection (AHI). The RV254/SEARCH010 study (clinicaltrials.gov NCT00796146) enrolls participants diagnosed in the earliest stages of AHI at the Thai Red Cross AIDS Research Center in Bangkok, Thailand (103). ART was offered to all participants as part of a separate protocol (NCT00796263). Participants were categorized into stages 1–5 of AHI as previously described (18, 56, 57) (Table 1): Stage 1 (S1)—positive HIV-1 RNA, non-reactive 4th-generation (4G) IA, non-reactive 3G IA; S2—positive HIV-1 RNA, reactive 4G IA, non-reactive 3G IA; S3—positive HIV-1 RNA, reactive 4G IA, reactive 3G IA, negative western blot (WB); S4—positive HIV-1 RNA, reactive 4G IA, reactive 3G IA, indeterminate WB; and S5—positive HIV-1 RNA, reactive 4G IA, reactive 3G IA, positive WB except p31. Matched plasma, peripheral blood mononuclear cells (PBMCs), and LNMCs from the time of diagnosis in AHI were analyzed. Long-term antibody levels were also measured in plasma samples collected after 48 weeks of ART as available. Samples from chronically infected, treatment-naïve individuals and healthy HIV^-^ individuals who were enrolled in the RV304/SEARCH013 study (NCT01397669) in Bangkok were analyzed for comparison. The sample size was determined based on the availability of samples from participants who consented to lymph node biopsy during the different stages of HIV-1 infection.

### HIV-1 DNA measurements

The frequencies of LNMCs harboring HIV-1 DNA were measured by real-time PCR using LTR-gag (for total) and Alu-gag (for integrated) primers as previously described (16, 104).

### Flow cytometric analysis

PBMCs were isolated from peripheral blood by Lymphoprep density centrifugation and cryopreserved in fetal bovine serum (FBS) supplemented with 10% dimethyl sulfoxide. For processing of lymph node samples, after removal of surrounding fat lymph node tissue was minced and passed through a 70 µm nylon strainer. Cells were washed and counted before being cryopreserved. Frozen PBMCs or LNMCs were thawed for phenotypic analysis by flow cytometry as allowed by recovered cell number. Cells were stained with Live/Dead for 10 minutes at room temperature (RT) and then incubated with antibodies targeting surface proteins for 20 minutes at 4°C. Cells were washed twice with wash buffer (PBS containing 2% FBS) before fixation. For panels measuring the expression of surface proteins only, cells were fixed in PBS containing 2% formaldehyde. For intracellular staining, cells were fixed and permeabilized with the Foxp3/Transcription Factor Staining Buffer Set. Cells were stained with antibodies recognizing intracellular targets for 30 minutes at RT followed by washes with Foxp3 permeabilization buffer and wash buffer, respectively. Cells were analyzed on an LSRII (BD Biosciences) and data were analyzed with FlowJo (FlowJo, LLC, Ashland, OR). Antibodies used for flow cytometry are provided in Table S1.

### HIV-flow assay

The HIV-flow assay was used to quantify and analyze the phenotype of cells expressing p24 protein (105). Briefly, CD4^+^ T cells were isolated by negative magnetic selection using the EasySep Human CD4^+^ T Cell Enrichment Kit (StemCell Technologies, Vancouver, Canada). Purity was typically >98%. 5–15 × 10^6^ CD4^+^ T cells were resuspended at 2 × 10^6^ cells/mL in RPMI +10% FBS with antiretroviral drugs added (200 nM raltegravir, 200 nM lamivudine). Cells were rested for 18 hours, stained with Live/Dead for 20 min at 4°C, and then incubated with antibodies targeting surface proteins in PBS + 4% human serum (Atlanta Biologicals, Flowery Branch, GA) for 20 min at 4°C. Cells were fixed and permeabilized for 45 minutes with the Foxp3/Transcription Factor Staining Buffer Set and then stained with antibodies targeting intracellular markers in FoxP3 Buffer for 45 minutes at RT. Cells were washed and resuspended in PBS for analysis on a BD FACS ARIA III (BD Biosciences).

### Measurement of plasma CXCL13

Plasma CXCL13 levels were measured using Luminex technology with a ProcartaPlex multiplex immunoassay (Assay MXH49YW) (Life Technologies Corporation, Carlsbad, CA, USA). Samples were run according to the manufacturer’s instructions and cytokine standards were provided by the manufacturer. A Bioplex-200 system was used to acquire samples, and the data were analyzed with the BioPlex Manager Software (Bio-Rad Laboratories, Hercules, CA, USA).

### Measurement of HIV-specific antibody levels

HIV-specific antibody levels were measured as previously described (18). High-binding half-area microplates (Grenier Bio-One, Kremsmünster, Austria) were coated with consensus CRF01_AE gp120 (Immune Technology Corp, New York, NY, USA) protein at a concentration of 1 µg/mL in PBS and incubated overnight at 4°C. Plates were washed 5× with wash buffer (PBS + 0.05% Tween 20) before blocking with PBS plus 10% (vol/vol) FBS for 1 hour at RT. After washing again, dilutions of plasma and standards were added in duplicate and incubated for 2 hours at RT. Anti-gp120/gp160 (CRF01_AE) (Immune Technology Corp., clone 26A4) was used for the standard curve. After subsequent washing, plates were incubated for 1 hour at RT with 1 µg/mL of biotin-conjugated anti-human IgG (Mabtech, clone MT78/145), or anti-mouse IgG (Mabtech) for the gp120 standard curve. Plates were then washed and incubated with streptavidin–horseradish peroxidase (Mabtech) for 1 hour at RT. After a final wash, 50 µL of TMB substrate (Sigma-Aldrich, St. Louis, MO) was added until the appearance of color, and the enzymatic reaction was stopped by adding 50 µL of 1M H_3_PO_4_. The absorbance was read at 450 nM using a Versamax Tunable Microplate Reader (Molecular Devices, LLC, San Jose, CA).

### ADCP assay

ADCP was measured as previously described (106). Briefly, gp120 CM235 (NIH HIV Reagent Program, Division of AIDS, NIAID, NIH: Human Immunodeficiency Virus 1 (HIV-1) CM235 gp120 Recombinant Protein, ARP-12816, contributed by NIAID, DAIDS) was biotinylated at a biotin to protein ratio of 50 following the manufacturer’s instructions (Thermo Scientific) and incubated with yellow-green streptavidin-fluorescent beads (Molecular Probes) for 2 hours at 37°C. 10 µL of a 100-fold dilution of beads–protein was incubated 2 hours at 37°C with 100 µL of 200-fold diluted plasma samples before the addition of THP-1 cells (20,000 cells per well; Millipore Sigma). After 18 hours of incubation at 37°C, the cells were fixed with a 4% formaldehyde solution (Tousimis) and fluorescence was evaluated on an LSRII (BD Biosciences). The phagocytic score was calculated by multiplying the percentage of bead-positive cells by the geo mean fluorescence intensity of the bead-positive cells and dividing by 10^4^.

### Infection of CEM.NKR_CCR5_ cell line with HIV-1 IMCs

The HIV-1 reporter viruses used were replication-competent infectious molecular clones (IMC) designed to encode the *env* genes of CM235 (subtype A/E; GenBank No. AF259954.1) as previously described (107). CEM.NKR_CCR5_ cells were infected with HIV-1 IMCs as previously described (107). Briefly, IMCs were titrated to achieve maximum infection within 48–72 hours post-infection as determined by detection of Luciferase activity and intra-cellular p24 expression. For each ADCC assay, we monitored the frequency of infected target cells by intracellular p24 staining. Assays performed using infected target cells were considered reliable if cell viability was ≥60% and the percentage of viable p24^+^ target cells on assay day was ≥20%.

### Luciferase ADCC assay

We utilized a modified version of the ADCC luciferase assay (108). Briefly, CEM.NKR_CCR5_ cells were infected with HIV-1 IMCs as described above and used as target cells. For effector cells, cryopreserved PBMC obtained by leukapheresis from an HIV-seronegative individual (Fc-gamma-Receptor IIIa 158 V/F heterozygous) were thawed the day before the assay and rested overnight in RPMI 1640 medium supplemented with antibiotics and 10% fetal bovine plasma (R10), and with recombinant human IL-15 at a concentration of 10 ng/mL. Effector and target cells (30:1 ratio) were plated in opaque 96-well half-area plates and co-cultured with serial dilutions of plasma. Each plasma sample was assayed at six dilutions, starting at a dilution of 1:50, with duplicate wells set up for each dilution. For the fourfold serial dilution scheme, plasma dilutions of 1:50, 1:200, 1:800, 1:3,200, 1:12,800, and 1:51,200 were used. Co-cultures were incubated for 6 hours at 37°C in 5% CO_2_ in IL-15 supplemented R10. The assay readout is luminescence intensity (measured in relative light units, RLUs) generated by surviving target cells that have not been lysed by the effector population in the presence of ADCC-mediating plasma Abs. The monoclonal Ab palivizumab (Synagis), which mediates ADCC (109) but is specific for the respiratory syncytial virus, and a cocktail of HIV-1 monoclonal Abs demonstrated to mediate ADCC (A32 [110], 2g12 [111], CH44 [112], and 7B2 [113]) were used as negative and positive controls, respectively. All mAbs were generated using IgG1 constant region containing alanine substitutions (S298A, E333A, and K334A) designed to enhance binding to Fc-gamma-receptor IIIa (FcγR3A) (114). A response was reported as positive if it was greater than 15 specific killings at the first two dilutions after subtracting the average % specific killing observed by testing a panel of 11 sera collected from geographically matched seronegative subjects. The ADCC antibody titer, defined as the last dilution of plasma capable of mediating ADCC in our *in vitro* assay, was calculated by interpolation of the dilution curve intersected by the positive cutoff of greater than 15% specific killing.

### Antibody binding assay

Ab binding to HIV-1-infected cells was measured as described previously (115, 116). Briefly, 7.5 × 10^5^ CM235 IMC-infected CEM.NKR_CCR5_ cells were incubated with 100-fold diluted human plasma for 2 hours at 37°C followed by surface staining with anti-IgG secondary-PECy7 (Biolegend, clone M1310G05) and anti-CD4–APC (Biolegend, clone OKT4) for 20 minutes at room temperature. Cells were then resuspended in 100 µL Cytofix/Cytoperm and incubated for 20 minutes at 4°C, followed by staining with anti-p24-FITC antibody (Beckman Coulter, clone KC57) for 25 minutes at room temperature. Cells were washed and resuspended in 125 µL PBS—1% paraformaldehyde. The samples were acquired within 24 hours using a BD Fortessa cytometer (BD Biosciences), and plasma binding was quantified as the percentage of target cells positive for anti-IgG secondary antibody after background subtraction.

### Statistics

Statistical analyses were performed using the Kruskal-Wallis test with Dunn’s multiple comparisons test to measure differences between stages of AHI or multiple cell populations or the Wilcoxon signed-rank test to measure differences between frequencies of CXCR3^−^ and CXCR3^+^ cells. Median values for groups are shown in Figs. Correlations were performed with the nonparametric Spearman test. Spearman correlations were corrected for a false discovery rate of 5% using the Benjamini-Hochberg procedure. The participant infected with a Clade B virus was excluded from the analysis of antibody correlations. One participant had an detectable viral load after 48 weeks of analysis and was excluded from the analysis of antibody correlations from this time point, three participants did not have samples after 48 weeks of ART for analysis. All statistical analyses were performed using GraphPad Prism (GraphPad Software Inc., La Jolla, CA) and significance was defined as *P* < 0.05 for two-sided testing.

## Data Availability

The data that support the findings of this study are available from the corresponding author upon reasonable request.

## SUPPLEMENTAL MATERIAL

Supplemental material jvi.01532-24-s0001.pdf Figures S1 to S12; Table S1.

ASM does not own the copyrights to Supplemental Material that may be linked to, or accessed through, an article. The authors have granted ASM a non-exclusive, world-wide license to publish the Supplemental Material files. Please contact the corresponding author directly for reuse.
