# Supplementary material for: Activation of CXCR3+ Tfh cells and B cells in lymph nodes during acute HIV-1 infection correlates with HIV-specific antibody development
Source: J Virol. 2025 Feb 11;99(3):e01532-24. doi: 10.1128/jvi.01532-24 (PMC11915809; doi:10.1128/jvi.01532-24)
Supplement: Supplemental material — Figures S1 to S12; Table S1. [file jvi.01532-24-s0001.pdf]

Supplemental Materials

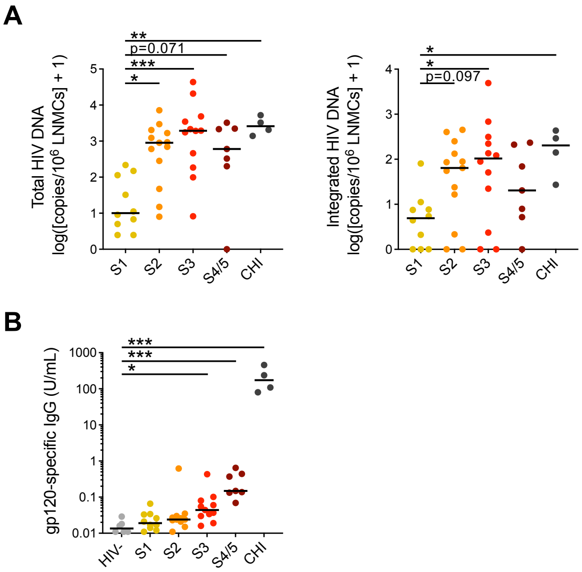

Figure S1. HIV DNA and antibody levels prior to ART initiation.

(A) Total and integrated HIV levels were measured in lymph node mononuclear cells (LNMCs) from prior to ART initiation in AHI. (B) Levels of gp120-specific IgG antibodies were measured in the plasma prior to ART initiation in AHI and in participants living without HIV. Changes during HIV infection were measured by a Kruskal-Wallis test with Dunn’s multiple comparison to participants in S1 (A) or HIV- participants (B). N=50 *p<0.05, **p<0.01, ***p<0.001

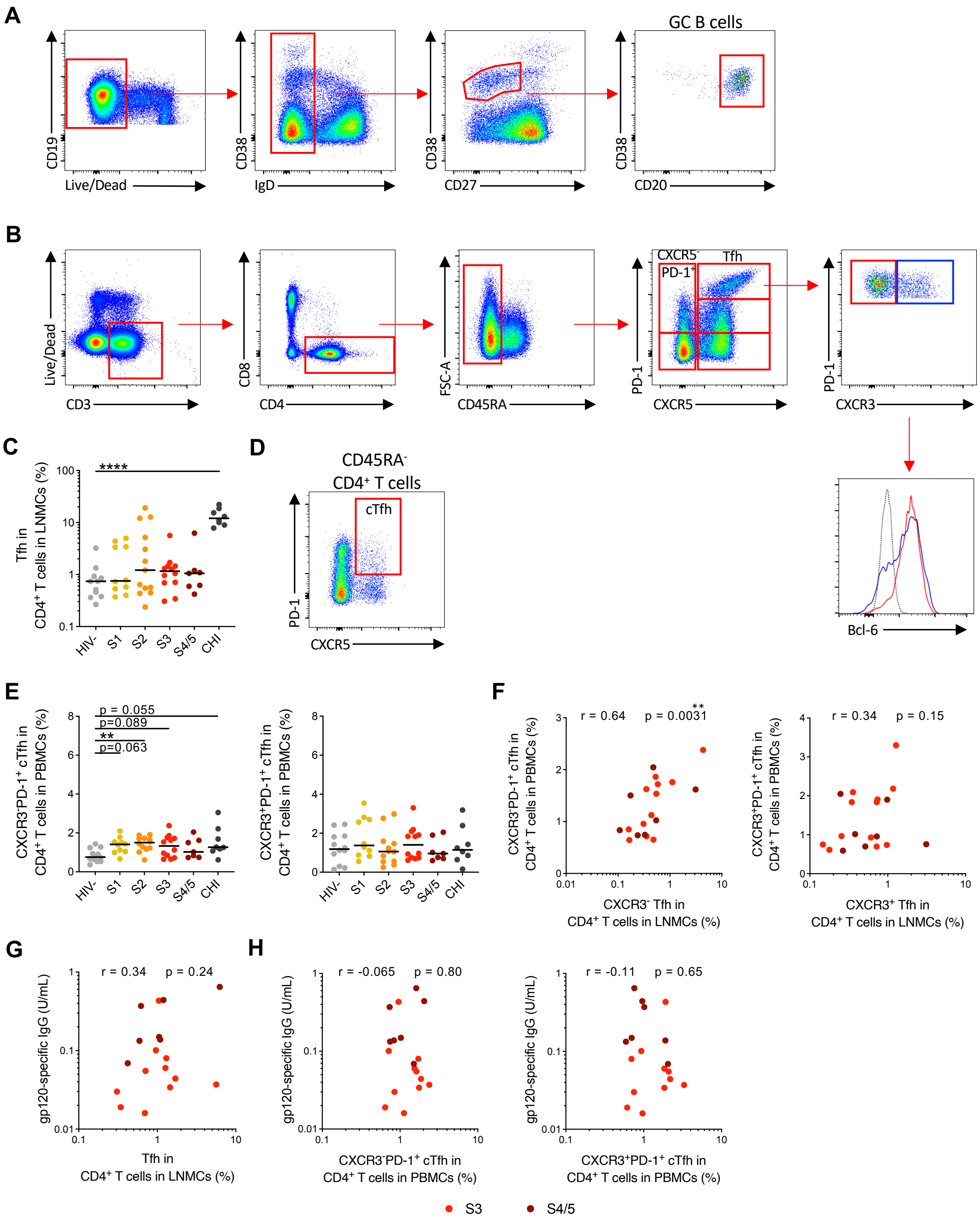

Figure S2. Total cTfh frequencies in the blood do not correlate with plasma HIV-specific antibody levels in acute HIV infection. (A) GC B cells were identified in LNMCs by flow cytometry. GC B cells were gated as IgD^{-}CD38^{+}CD27^{lo}CD20^{hi} cells within CD19^{+} live B cells. Representative gating from a participant in S4 is shown. (B) CD4^{+} T cell populations were identified in LNMCs by flow cytometry. Tfh were gated as CXCR5^{+}PD-1^{hi} cells within

$CD45RA^-CD4^+$ live T cells. Gating of $CXCR3^-$ and $CXCR3^+$ populations within Tfh cells is shown. High bcl-6 expression in $CXCR3^-$ (red line) and $CXCR3^+$ (blue line) Tfh cells is shown compared to bcl-6^- $CXCR5^+PD\text{-}1^-$ cells (dotted line). Representative gating from a participant in S2 with a relatively high frequency of Tfh (5% of $CD4^+$ T cells) is shown. (C) Frequencies of Tfh ($CXCR5^+PD\text{-}1^{hi}$ $CD4^+$ T cells) cells were measured in LNMCs of participants in the different stages of AHI or CHI by flow cytometry. (D) Circulating Tfh (cTfh) cells were gated as $CXCR5^+PD\text{-}1^+$ cells within the $CD45RA^-$ $CD4^+$ T cell population as shown. (E) $CXCR3^-$ and $CXCR3^+$ cTfh cell frequencies in HIV^- controls and participants in the different stages of AHI. (F) Correlations between $CXCR3^-$ and $CXCR3^+$ Tfh cells in lymph nodes with respective $CXCR3^-$ or $CXCR3^+$ cTfh in the blood in S3-5 of AHI. (G) Correlation between the frequencies of Tfh cells in the lymph nodes with levels of gp120-specific IgG antibodies int eh plasma. (F) Correlations of $CXCR3^-$ or $CXCR3^+$ cTfh frequencies in the blood with levels of gp120-specific IgG antibodies in the plasma. Changes in cell populations during HIV infection were measured by a Kruskal-Wallis test with Dunn's multiple comparison to HIV^- controls. Correlations were measured by Spearman correlation. Correlations that remained significant after correction for a false discovery rate (FDR) of 5% are indicated by asterisks (*). N=62 **$p<0.01$, ****$p<0.0001$

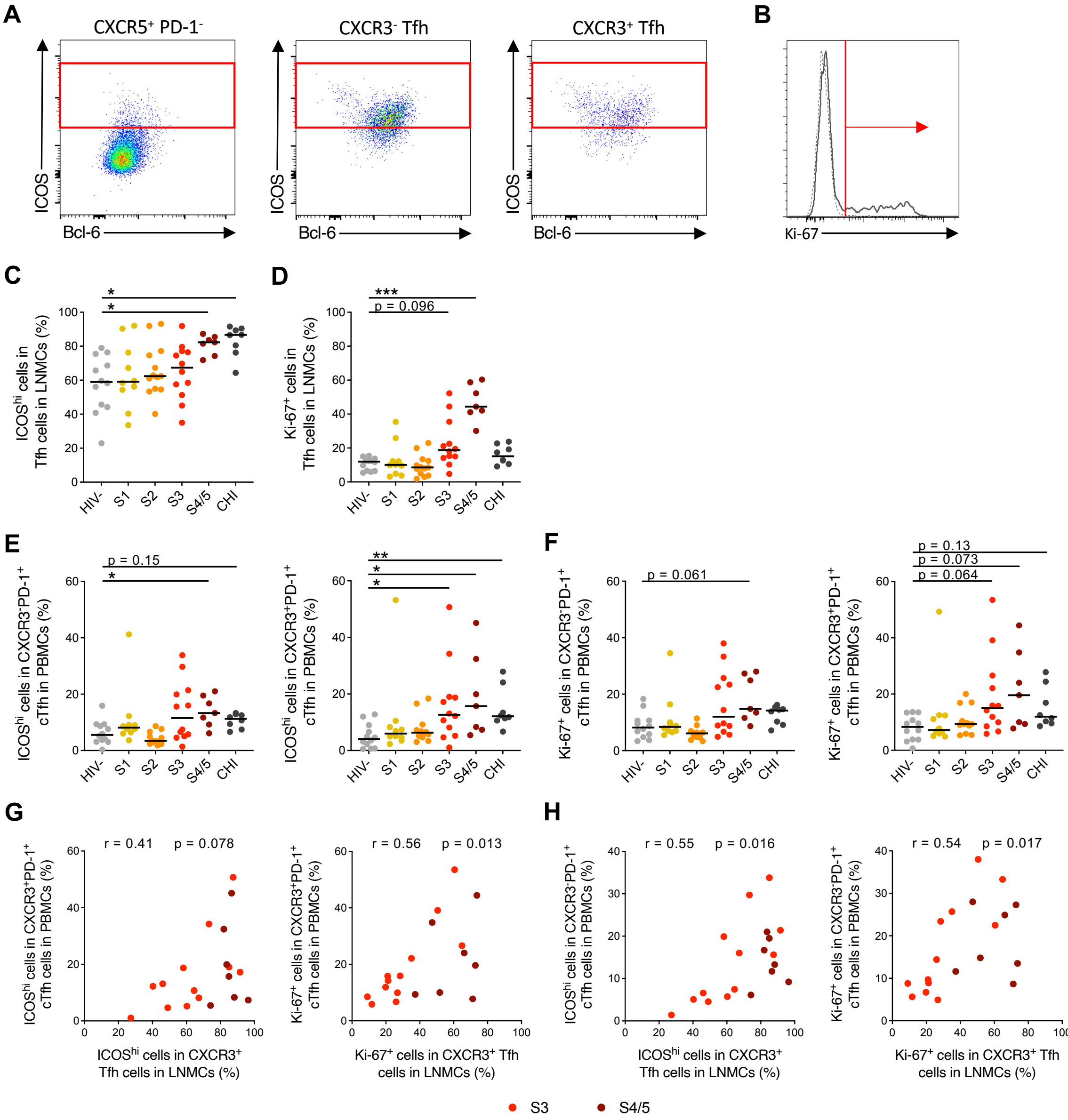

Figure S3. cTfh in the blood show increased activation and proliferation during acute HIV infection. (A) Gating of ICOS^{hi} cells was based on ICOS expression within CXCR5^{+}PD-1^{-} non-Tfh CD4^{+} T cells. Representative gating of ICOS^{hi} cells within CXCR5^{+}PD-1^{-} CD4^{+} T cells, CXCR3^{-} Tfh, and CXCR3^{+} Tfh cells in the lymph node is shown for the same participant in Supplemental Figure 2. (B) Gating of Ki-67^{+} cells is shown for CXCR3^{-} Tfh in the lymph node of the same participant from S2 of AHI. Frequencies of ICOS^{hi} cells (C) and Ki-67^{+} cells (D) within CXCR3^{-}PD-1^{+} and CXCR3^{+}PD-1^{+} cTfh populations were measured by flow cytometry in

matching PBMCs from participants prior to ART initiation in different stages of AHI or in CHI. Changes in frequencies were measured by a Kruskal-Wallis test with Dunn's multiple comparison to HIV^- controls. Correlations between the frequency of Ki-67^+ or $ICOS^{hi}$ cells within $CXCR3^+$ Tfh in lymph nodes and those within $CXCR3^+$PD-1^+ (E) or $CXCR3^-$PD-1^+ (F) cTfh in the blood. Correlations were measured by Spearman correlation. No correlations remained significant after correction for a FDR of 5%. N=62 *$p<0.05$, **$p<0.01$

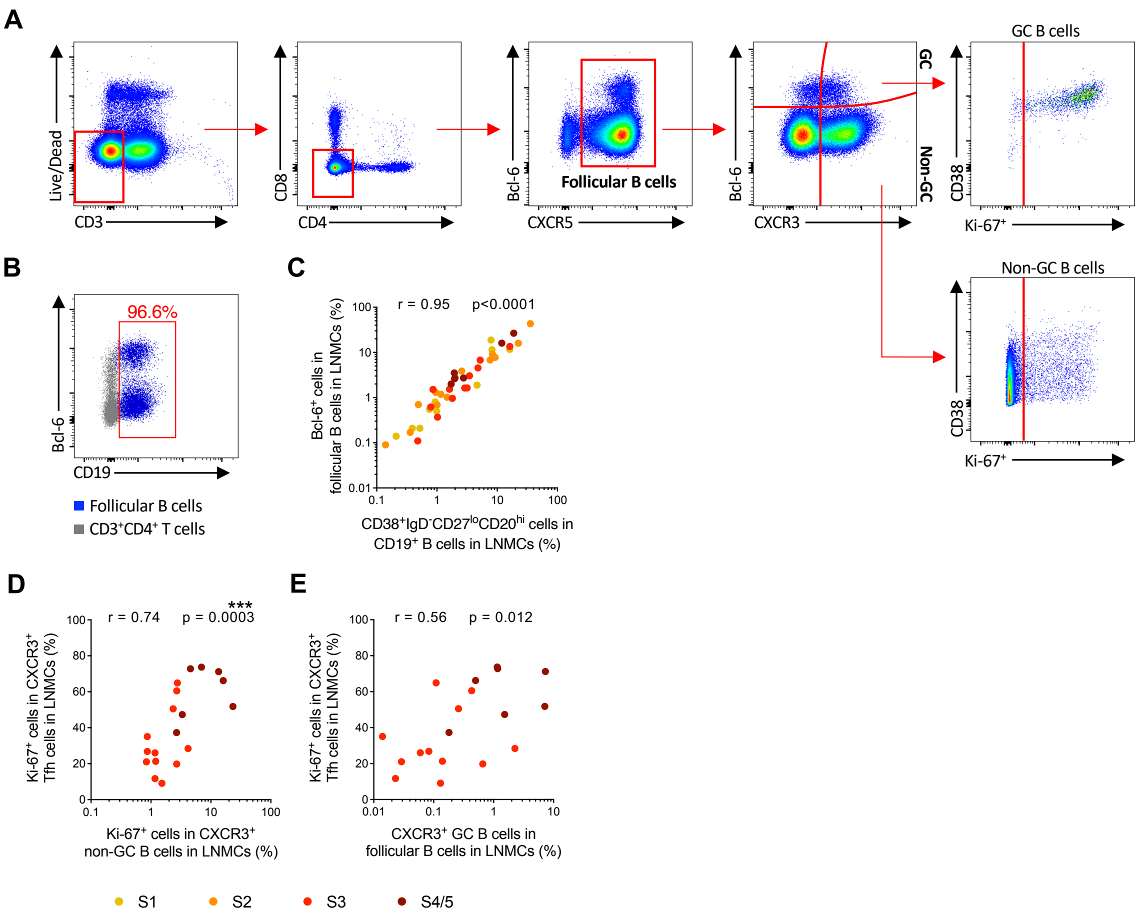

Figure S4. Correlations between follicular B cell populations and Tfh activation in lymph nodes in acute HIV infection. (A) Gating strategy for CXCR5$^+$ follicular B cells. CXCR5$^+$ cells were gated from CD3$^-$CD4$^-$CD8$^-$ cells. CXCR5$^+$ cells were further gated on Bcl-6 to identify Bcl-6$^-$ non-GC B cells and Bcl-6$^+$ GC B cells, and on CXCR3 expression. The frequency of Ki-67$^+$ cells was then determined. Representative gating for a participant from S4 with a near median frequency of CXCR3$^+$ GC B cells (1.53%) is shown. (B) Representative graph showing CD19 expression on CXCR5$^+$CD3$^-$CD4$^-$CD8$^-$ follicular B cells (blue) compared to CD3$^+$CD4$^+$ T cells (grey). (C) Correlation between the frequency of germinal center B cells identified by gating on the frequency of IgD$^-$CD38$^+$CD27^{lo}CD20^{hi} cells within CD19$^+$ live B cells and the frequency of Bcl-6$^+$ cells within CXCR5$^+$CD3$^-$CD4$^-$CD8$^-$ cells. (D) Correlation between the frequency of Ki-67$^+$ cells within CXCR3$^+$ non-GC B cells and Ki-67$^+$ cells within CXCR3$^+$ Tfh

cells. (E) Correlation between the frequency of $CXCR3^+$ GC B cells within follicular B cells and $Ki\text{-}67^+$ cells within $CXCR3^+$ Tfh cells. Correlations were measured by Spearman correlation. Correlations that remained significant after correction for a FDR of 5% are indicated by asterisks (*). N=42 ***$p<0.001$

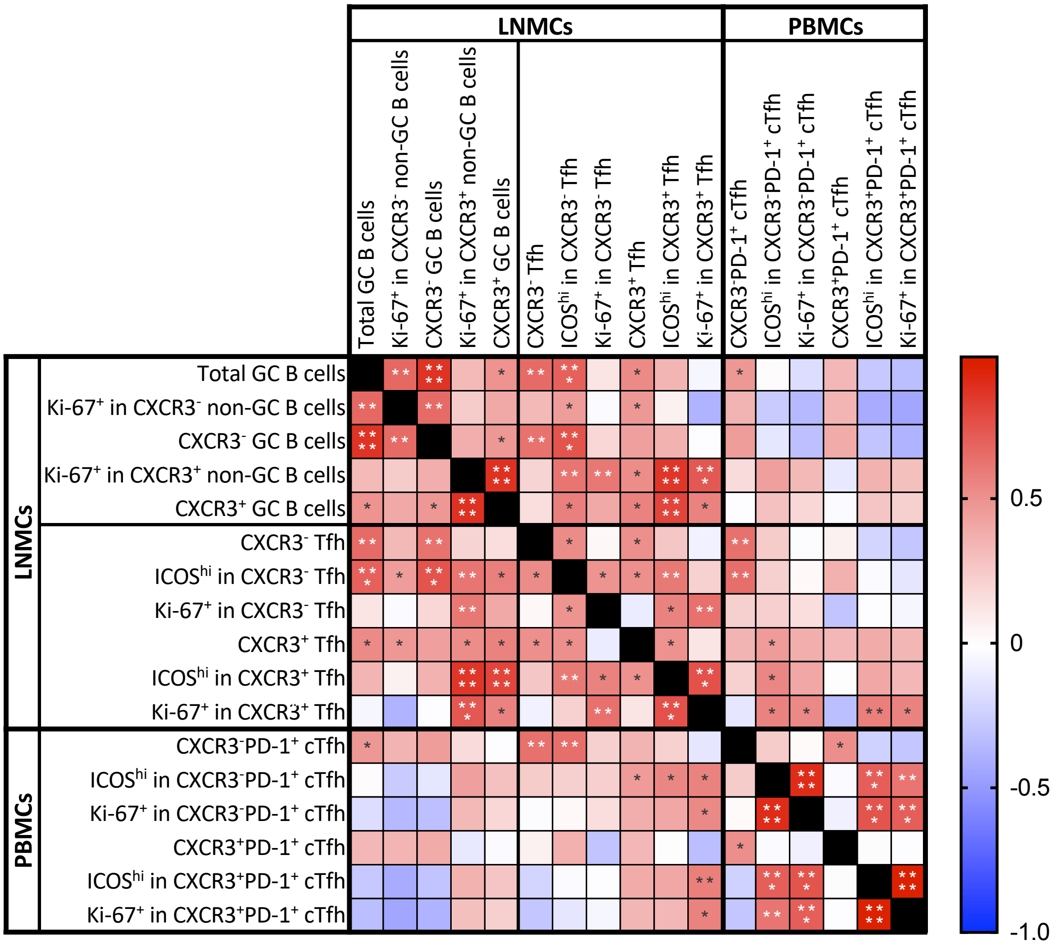

Figure S5. Correlation matrix for Tfh and B cell populations. A heatmap shows the Spearman r values for correlations between Tfh and B cell populations in the lymph nodes and cTfh populations in the blood at the time of diagnosis for participants who initiated treatment in S3-5 of AHI. All correlations were measured by Spearman correlation. Correlations that remained significant after correction for a FDR of 5% are indicated by white asterisks (*). N=18 *p<0.05, **p<0.01, ***p<0.001, ****p<0.0001

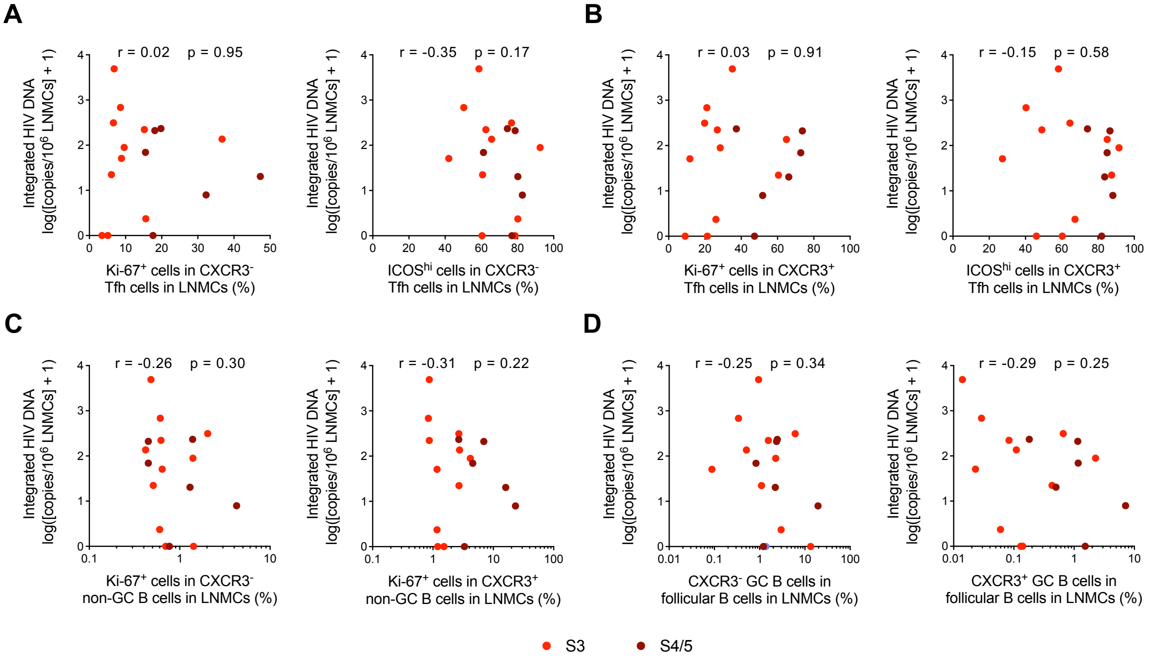

Figure S6. Correlations with integrated HIV DNA levels in the lymph nodes during AHI. Correlations between integrated HIV DNA levels in LNMCs and the frequencies of Ki-67^{+} or ICOS^{hi} CXCR3^{-} Tfh (A), Ki-67^{+} or ICOS^{hi} CXCR3^{+} Tfh (B), Ki-67^{+} CXCR3^{-} or CXCR3^{+} non-GC B cells (C), and CXCR3^{-} or CXCR3^{+} GC B cells (D) in the lymph nodes during AHI are shown. N=16

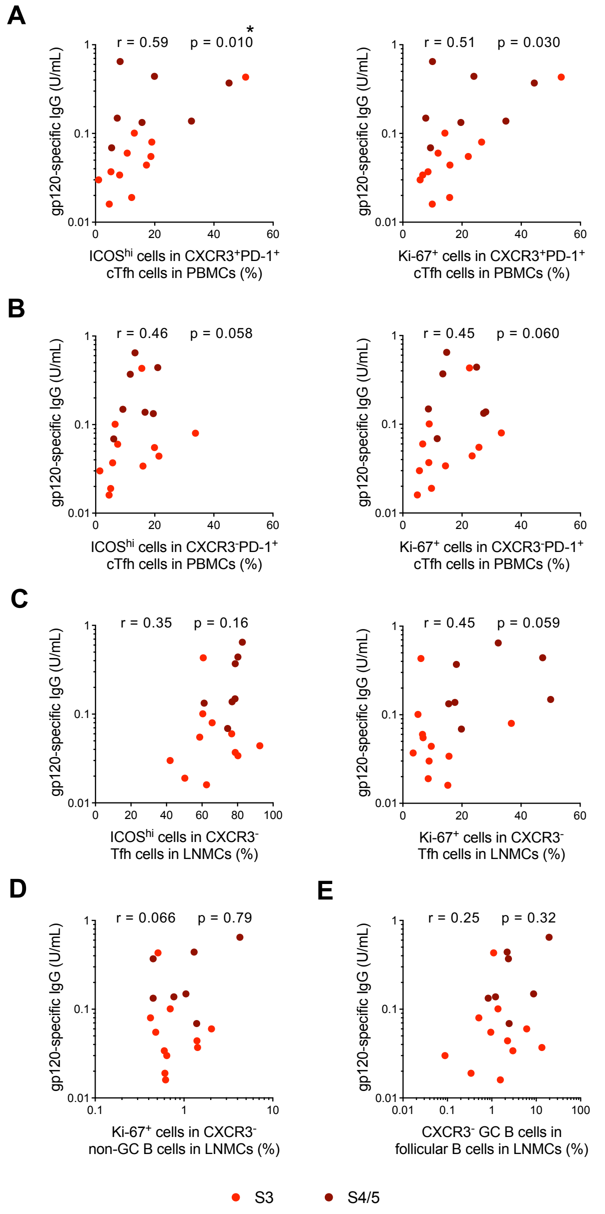

Figure S7. Frequencies of activated CXCR3$^-$ Tfh and B cells do not significantly correlate with plasma gp120-specific antibody levels in acute HIV infection. Correlations between plasma gp120-specific antibody levels at the time of diagnosis and the frequency of ICOS^{hi} or Ki-67$^+$ cells in CXCR3$^+$PD-1$^+$ cTfh (A), ICOS^{hi} or Ki-67$^+$ cells in CXCR3$^-$PD-1$^+$ cTfh (B), CXCR3$^-$ Tfh (C), CXCR3$^-$ non-GC B cells (D), and CXCR3$^-$ GC B cells (E). All correlations were measured by Spearman correlation. Correlations that remained significant after correction for a FDR of 5% are indicated by asterisks (*). N=18 *$p<0.05$

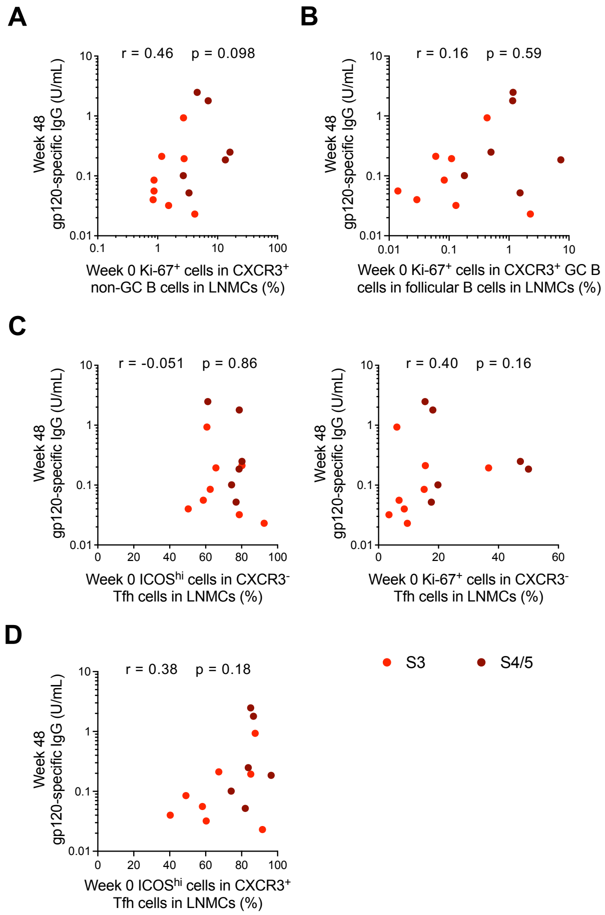

Figure S8. Correlations with HIV-specific antibody levels after 48 weeks of ART. gp120-specific antibodies were measured in the plasma 48 weeks after ART initiation, and correlations with the frequencies of Ki-67^{+} within CXCR3^{+} non-GC B cells (A), total CXCR3^{+} GC B cells (B), Ki-67^{+} and ICOS^{hi} cells within CXCR3^{-} Tfh (C), and ICOS^{hi} cells within CXCR3^{+} Tfh in lymph node during AHI are shown. All correlations were measured by Spearman correlation. No correlations remained significant after correction for a FDR of 5%. N=14

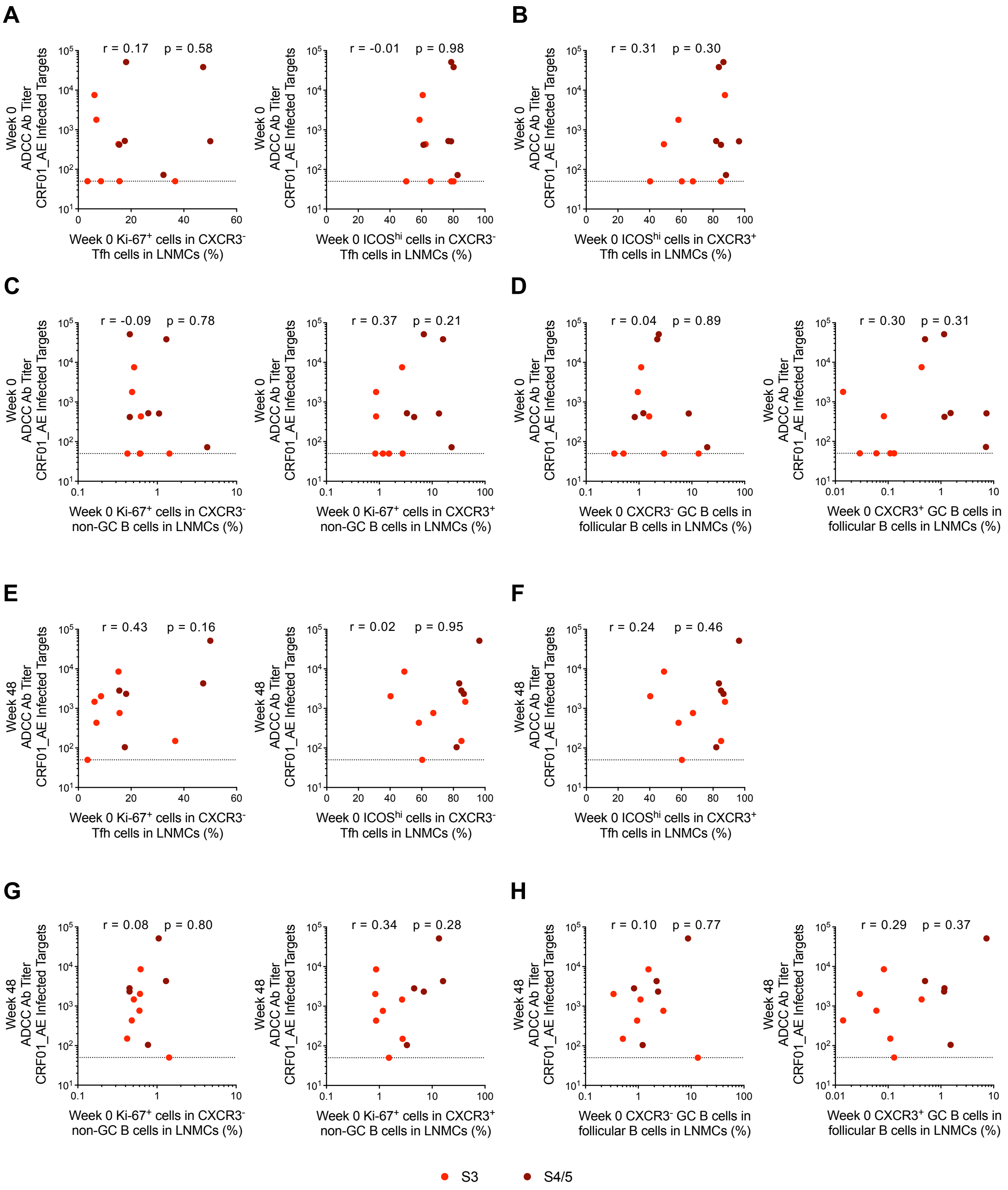

Figure S9. Correlations with ADCC titer pre-ART and after 48 weeks of ART. Antibody dependent cellular cytotoxicity (ADCC) responses of antibodies from plasma collected pre-ART or after 48 weeks of ART were measured using target cells infected with CRF01_AE virus. Correlations between ADCC antibody titers pre-ART and the frequencies of Ki-67^{+} or ICOS^{hi} CXCR3^{-} Tfh (A), Ki-67^{+} CXCR3^{+} Tfh (B), Ki-67^{+} CXCR3^{-} or CXCR3^{+} non-GC B cells (C),

and $CXCR3^-$ or $CXCR3^+$ GC B cells (D) in the lymph nodes during AHI are shown. Correlations between ADCC antibody titers after 48 weeks of ART and the frequencies of Ki-67^+ or $ICOS^{hi}$ $CXCR3^-$ Tfh (E), Ki-67^+ $CXCR3^+$ Tfh (F), Ki-67^+ $CXCR3^-$ or $CXCR3^+$ non-GC B cells (G), and $CXCR3^-$ or $CXCR3^+$ GC B cells (H) in the lymph nodes during AHI are shown. N=13 for Week 0; N=12 for Week 48

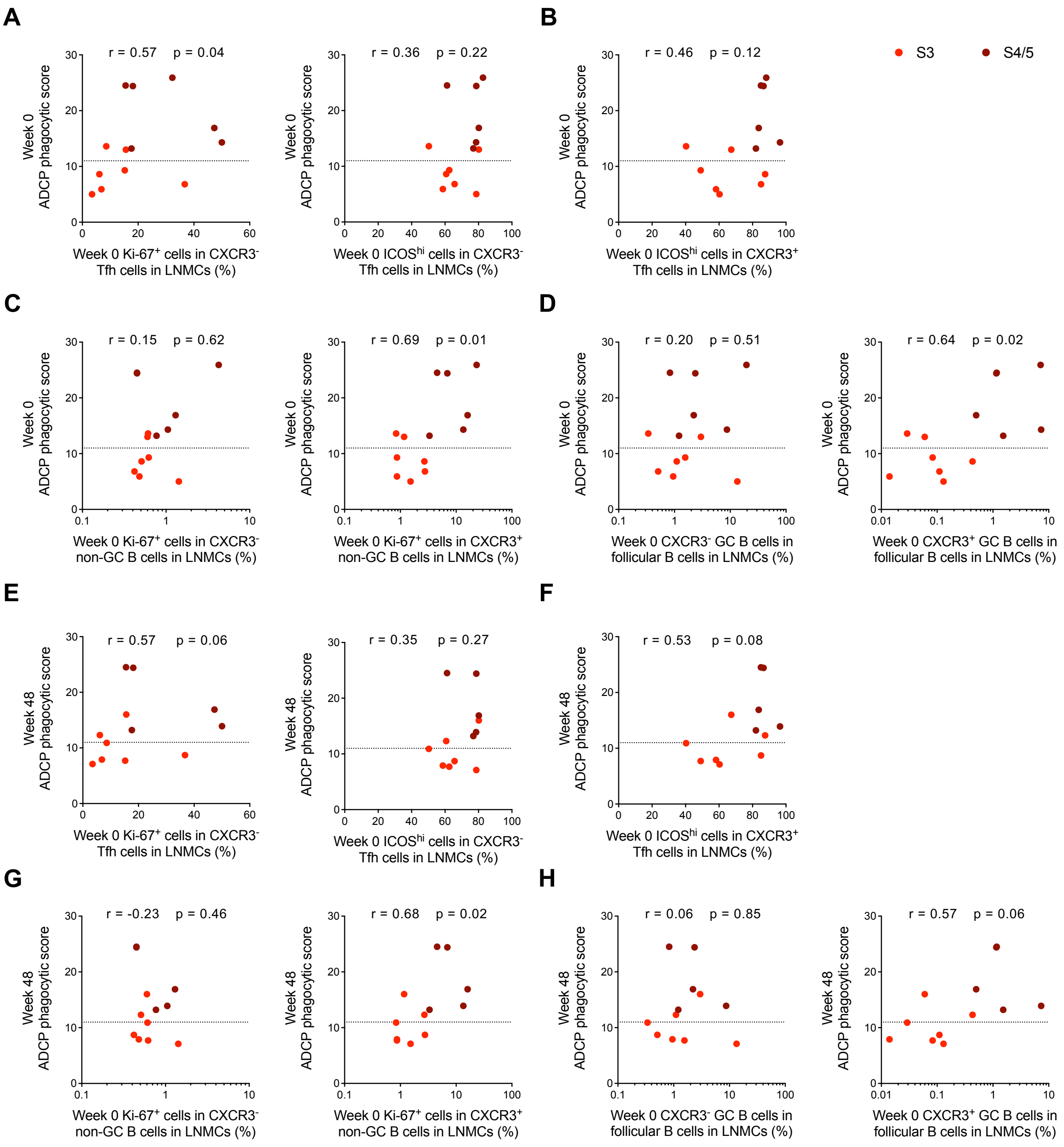

Figure S10. Correlations with ADCP phagocytic scores pre-ART and after 48 weeks of ART. Antibody dependent cellular phagocytosis (ADCP) responses of antibodies from plasma collected pre-ART or after 48 weeks of ART were measured using CRF01_AE gp120 conjugated beads. Correlations between ADCP phagocytic scores pre-ART and the frequencies of Ki-67$^+$ or ICOS^{hi} CXCR3$^-$ Tfh (A), Ki-67$^+$ CXCR3$^+$ Tfh (B), Ki-67$^+$ CXCR3$^-$ or CXCR3$^+$ non-GC B cells (C), and CXCR3$^-$ or CXCR3$^+$ GC B cells (D) in the lymph nodes during AHI are

shown. Correlations between ADCP phagocytic scores after 48 weeks of ART and the frequencies of Ki-67^{+} or $ICOS^{hi}$ $CXCR3^{-}$ Tfh (E), Ki-67^{+} $CXCR3^{+}$ Tfh (F), Ki-67^{+} $CXCR3^{-}$ or $CXCR3^{+}$ non-GC B cells (G), and $CXCR3^{-}$ or $CXCR3^{+}$ GC B cells (H) in the lymph nodes during AHI are shown. N=13 for Week 0; N=12 for Week 48

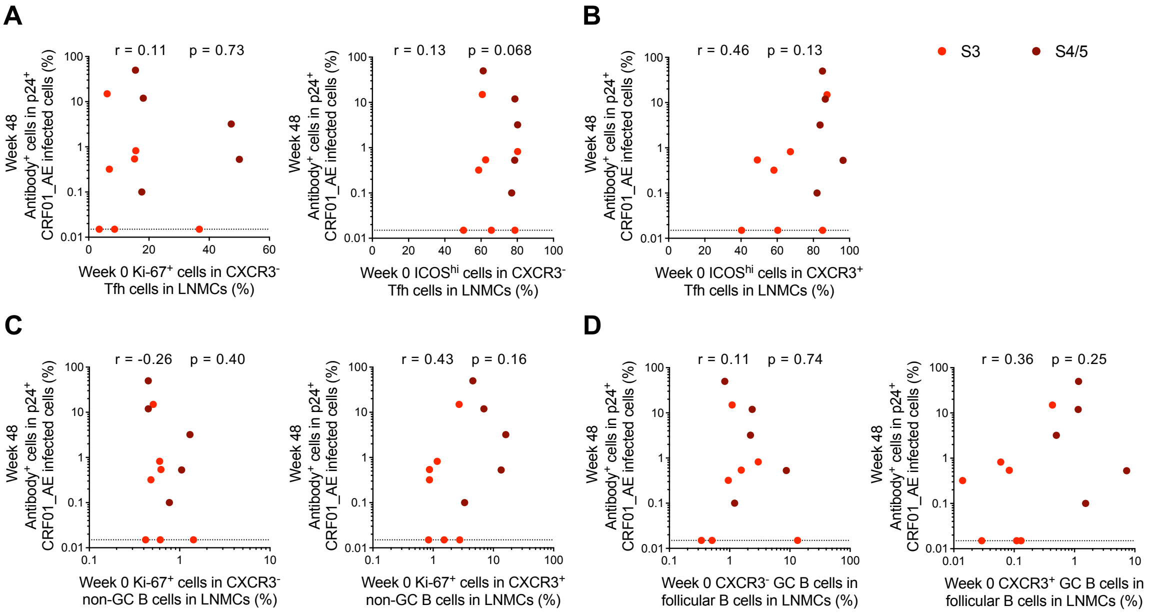

Figure S11. Correlations with antibody binding to CRF01_AE infected cells after 48 weeks of ART. Antibody binding activity was measured as the percent of $p24^+$ CRF01_AE infected cells stained with anti-IgG secondary antibody after subtraction of background. Correlations between frequencies of $p24^+$ cells bound by antibody from plasma after 48 weeks and the frequencies of Ki-67^+ or ICOS^{hi} CXCR3^- Tfh (A), Ki-67^+ CXCR3^+ Tfh (B), Ki-67^+ CXCR3^- or CXCR3^+ non-GC B cells (C), and CXCR3^- or CXCR3^+ GC B cells (D) in the lymph nodes during AHI are shown. N=12

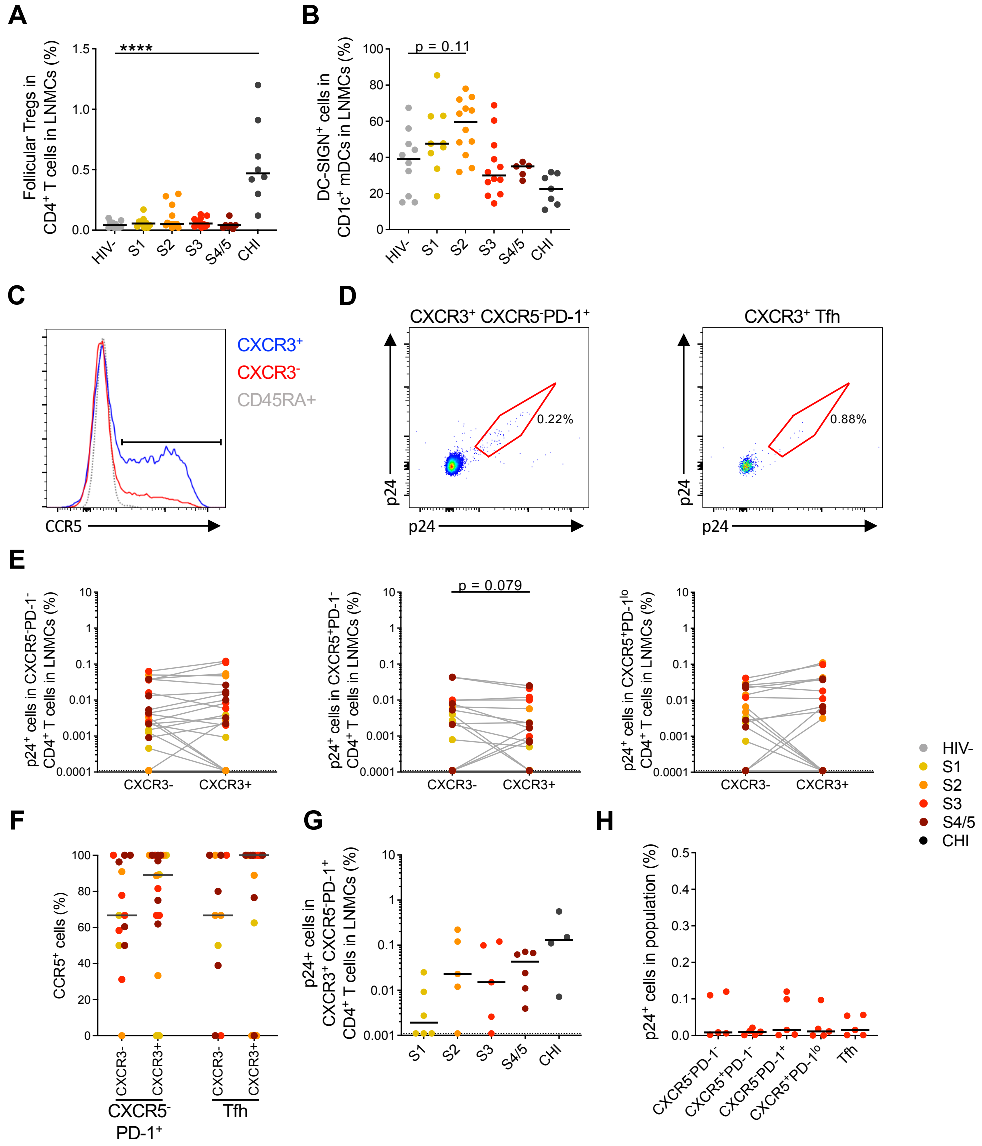

Figure S12. Infection of CD4$^+$ T cell subsets.

(A) The frequency of follicular regulatory T (Treg) cells (CD45RA$^-$CXCR5$^+$PD-1^{hi}Foxp3$^+$CD127^{lo} cells) was measured in CD4$^+$ T cells in LNMCs from participants in different stages of AHI or CHI by flow cytometry. (B) The frequency of DC-SIGN expressing cells with CD1c$^+$ myeloid dendritic cells (mDCs; Lineage$^-$HLA-DR$^+$CD11c$^+$CD1c$^+$ cells) was measured by flow cytometry in LNMCs from participants in different stages of AHI or in CHI. Changes in frequencies between stages were measured by a Kruskal-Wallis test with Dunn's multiple comparison to HIV$^-$ controls. (C) Gating of CCR5$^+$ cells is shown for CXCR3$^-$ and CXCR3$^+$

populations of $CXCR5^{-}PD\text{-}1^{+}$ $CD4^{+}$ T cells in the lymph node of a participant from S5 of AHI. (D) p24-producing HIV-infected $CD4^{+}$ T cells were measured in LNMCs by HIV-Flow. Representative gating of doubly stained $p24^{+}$ cells within $CXCR3^{+}$ $CXCR5^{-}PD\text{-}1^{+}$ $CD4^{+}$ T cells and $CXCR3^{+}$ Tfh from one participant is shown. (E) Comparisons of the frequency of $p24^{+}$ cells between $CXCR3^{-}$ and $CXCR3^{+}$ populations are shown for $CXCR5^{-}PD\text{-}1^{-}$, $CXCR5^{+}PD\text{-}1^{-}$, and $CXCR5^{+}PD\text{-}1^{+}$ $CD4^{+}$ T cells. Differences were measured by a Wilcoxon test. (F) Frequencies of $CCR5^{+}$ cells with $p24^{+}$ cells with the given non-Tfh and Tfh phenotypes. (G) Frequencies of $p24^{+}$ cells within $CXCR3^{+}$ $CXCR5^{-}PD\text{-}1^{+}$ non-Tfh in LNMCs from participants in AHI and CHI. Changes in frequencies were measured by a Kruskal-Wallis test with Dunn's multiple comparison between AHI stages. (H) Frequencies of $p24^{+}$ cells within different $CXCR3^{+}$ memory $CD4^{+}$ T cell populations in LNMCs from participants in S3 of AHI. Changes in frequencies were measured by a Kruskal-Wallis test with Dunn's multiple comparison to Tfh. N=56 for *A-B*, N=26 for *E-G*, N=5 for *H*

Table S1.

B cell panel			
Target	Fluorophore	Company	Clone
CD10	PE/Dazzle 594	BioLegend	HI10a
CD19	BV785	BioLegend	HIB19
CD20	BV650	BioLegend	2H7
CD21	PerCP Cy5.5	BioLegend	Bu32
CD27	BV421	BD	M-T271
CD3	Alexa Fluor 700	BD	UCHT1
CD4	BUV496	BD	SK3
IgD	BV605	BioLegend	IA6-2
IgG	APC	BioLegend	M1310G05
IgM	FITC	BioLegend	MHM-88
Ki-67	BUV395	BD	B56
PDL1	PE Cy7	BioLegend	29E.2A3
CD38	PE	BioLegend	HIT2
CD40	APC Cy7	BioLegend	5C3
**$CD4^+$ T cell panel**			
Target	Fluorophore	Company	Clone
Bcl-6	PE	BD	K112-91
CCR7	PE CF594	BD	150503
CD127	BV650	BioLegend	A019D5
CD25	BV711	BioLegend	BC96
CD3	Alexa Fluor 700	BD	UCHT1
CD38	BUV737	BD	HB7
CD4	BUV496	BD	SK3
CD45RA	APC H7	BD	HI100
CD8	BV785	BioLegend	RPA-T8
CXCR3	BV421	BioLegend	G025H7
CXCR4	BV605	BioLegend	12G5
CXCR5	AF647	BD	RF8B2
Foxp3	Alexa488	eBioscience	PCH101
ICOS	PerCP Cy5.5	BioLegend	C398.4A
Ki-67	BUV395	BD	B56
PD-1	PE Cy7	BioLegend	EH12.2H7
**HIV-Flow panel**			
Target	Fluorophore	Company	Clone
p24	RD1	Beckman Coulter	KC57
p24	APC	MediMabs	28B7
CD4	APC-H7	BD	RPA-T4
CD45RA	Alexa Fluor 700	BD	HI100
CXCR5	BB515	BD	RF8B2
CXCR3	PerCP-Cy5.5	BD	1C6/CXCR3
Ki-67	PE-Cy7	BD	B56
CCR5	BV421	BD	2D7/CCR5
PD-1	BV605	BioLegend	EH12.2H7
ICOS	BV785	BioLegend	C398.4
